# Multiarmed DNA jumper and metal-organic frameworks–functionalized paper-based bioplatform for small extracellular vesicle–derived miRNAs assay

**DOI:** 10.1186/s12951-024-02546-w

**Published:** 2024-05-22

**Authors:** Xiaopei Qiu, Huisi Yang, Man Shen, Hanqing Xu, Yingran Wang, Shuai Liu, Qian Liu, Minghui Sun, Zishan Ding, Ligai Zhang, Jun Wang, Taotao Liang, Dan Luo, Mingxuan Gao, Ming Chen, Jing Bao

**Affiliations:** 1 Department of Clinical Laboratory Medicine, Southwest Hospital, Third Military Medical University (Army Medical University), Chongqing, 400038 P. R. China; 2 Key Laboratory for Biorheological Science and Technology of Ministry of Education, State and Local Joint Engineering Laboratory for Vascular Implants, Bioengineering College of Chongqing University, Chongqing, 400044 PR China; 3 Chongqing Sports Medicine Center, Department of Orthopedic Surgery, Department of Clinical Laboratory Medicine, Southwest Hospital, the Third Military Medical University, Chongqing, 400038 P.R. China; 4 Department of Biological and Environmental Engineering, Cornell University, Ithaca, NY 14853-5701 USA; 5 College of Pharmacy and Laboratory Medicine, Third Military Medical University (Army Medical University, Chongqing, 400038 China

**Keywords:** Metal–organic frameworks, Multiarmed DNA tetrahedral jumpers, Small extracellular vesicle–derived microRNAs, Wireless Biosensor, Point-of-care diagnosis

## Abstract

Small extracellular vesicle–derived microRNAs (sEV-miRNAs) have emerged as promising noninvasive biomarkers for early cancer diagnosis. Herein, we developed a molecular probe based on three-dimensional (3D) multiarmed DNA tetrahedral jumpers (mDNA-Js)-assisted DNAzyme activated by Na^+^, combined with a disposable paper-based electrode modified with a Zr-MOF–rGO–Au NP nanocomplex (ZrGA) to fabricate a novel biosensor for sEV-miRNAs Assay. Zr-MOF tightly wrapped by rGO was prepared via a one-step method, and it effectively aids electron transfer and maximizes the effective reaction area. In addition, the mechanically rigid, and nanoscale-addressable mDNA-Js assembled from the bottom up ensure the distance and orientation between fixed biological probes as well as avoid probe entanglement, considerably improving the efficiency of molecular hybridization. The fabricated bioplatform achieved the sensitive detection of sEV-miR-21 with a detection limit of 34.6 aM and a dynamic range from100 aM to 0.2 µM. In clinical blood sample tests, the proposed bioplatform showed results highly consistent with those of qRT-PCRs and the signal increased proportionally with the NSCLC staging. The proposed biosensor with a portable wireless USB-type analyzer is promising for the fast, easy, low-cost, and highly sensitive detection of various nucleic acids and their mutation derivatives, making it ideal for POC biosensing.

**Supplementary Information:**

The online version contains supplementary material available at 10.1186/s12951-024-02546-w.

## Introduction

Malignant tumors pose a considerable threat to human health. For precision medication, proactive health management is vital for early detection, treatment, predisease intervention, dynamic monitoring, and personalized treatment [1–3]. Liquid biopsy can identify cancer-associated biomarkers in body fluids and is a promising alternative for cancer diagnosis. Liquid biopsy employs noninvasive sampling and overcomes tumor heterogeneity issues. Further, it is easy to use and has a short time frame and high repeatability; it also dynamically reflects genetic information of the tumor [4, 5]. MicroRNAs (miRNAs) help in regulating pathological processes, making them good biomarkers for diagnosing and predicting various diseases [6–8]. MiRNAs derived from small extracellular vesicles (sEVs, 30–150 nm) are better than free miRNAs in blood because they contain more miRNA [9–12]. Furthermore, miRNAs are naturally encapsulated by sEVs, enabling them to circulate stably and resist RNase degradation, multiple freeze–thaw cycles, and extreme pH values [13–15]. Previous studies have shown that sEV-derived miRNA (sEV-miRNA) is a promising candidate for lung cancer diagnosis and prognostic evaluation [16, 17]. More than 30 miRNAs are upregulated in the exosomes of lung cancer patients. MiR-21, miRNA-155, and let-7b can be used to diagnose lung cancer recurrence, assess progression-free survival, and diagnose diseases [18]. 

Till date, exo-miRNAs are detected mainly by quantitative reverse transcription polymerase chain reactions (RT-qPCRs) and next-generation sequencing (NGS) [19–21]. However, such methods have some drawbacks, including high costs, false-positive amplifications, large sample volumes, and time-consuming processing steps, which have limited their onsite applications. Electrochemistry is a cost-effective, portable, sensitive, user-friendly, and quick-response technique employed in various point-of-care (POC) sensors with exceptional color resistance and ease of miniaturization [22–27]. However, it produces thin, single-dimensional bioactive layers on standard electrodes, inhibiting the early detection of nucleic acids. In our previous research, we developed a portable bioelectrode that employ a paper-based electrochemical technology [28]. To enhance the analytical sensitivity of biosensors, it is crucial to regulate their interfacial properties, which are influenced by the nanostructure of the interface and the coupling of the attached biomolecules [28, 29]. 

To enhance electron transport in biosensors, the electrodes of the biosensors must have uniformly controllable interfaces. The interfaces must meet the criteria for controllable morphology and the uniform dispersion of active sites. Among the nanomaterials, metal–organic frameworks (MOFs) are widely used in biomedical sensors owing to their unique properties, including high surface area, porosity, biodegradability, and chemical stability, which enhance the biosensing sensitivity and performance [30–34]. MOFs containing chemical moieties, such as amino or azide groups, enable precise biomolecule grafting to produce biofunctional materials [35, 36]. Zirconium-based MOFs (Zr-MOFs), the most prominent member of the UiO family, have excellent porosities and surface areas; thus, they contain a considerable number of signal molecules. Zr-MOFs can selectively immobilize DNA molecules owing to the strong Zr–O–P bonds formed with phosphate groups [37–39]. However, the low load capacities and slow responsive processes of Zr-MOFs have limited their applications [40]. Recently, graphene-based materials have been combined with MOFs to enhance their conductivities and stabilities and prevent restacking. Such combination has several advantages, including guiding the MOF growth, reducing conductivity limitations, and minimizing coordination bonding and performance issues. In situ growth exploits the oxygen groups on graphene oxide (GO)/reduced GO (rGO) surfaces to achieve uniform MOF growth, thereby saving time and enhancing adhesion [41, 42]. 

The sensitivity of nucleic acid detection can also be influenced by the coupling of different biomolecules on the sensing interface. It can be enhanced using a three-dimensional (3D) DNA tetrahedron (DNA-T) structure, which allows for better spatial control and probe accessibility compared to one-layered DNA probes, which may entangle at high concentrations or long strands [43–45]. Additionally, DNA-Ts undergo favorable self-assembly and exhibit mechanical rigidity and structural stability, which facilitate the accurate identification of units and specific orientations, improving the selectivity and reproducibility of trace nucleic acid detection [46, 47]. 

Owing to the highly sensitive and accurate detection of sEV-miRNAs by the synergistic effects of the Zr-MOF/rGO nanocomplex and multiarmed DNA tetrahedral jumper (mDNA-J), herein, we developed a Zr-MOF-rGO-Au (ZrGA)/mDNA-J portable bioplatform. In this bioplatform, the ZrGA nanocomplex was modified on the surface of a screen-printed carbon electrode (SPCE), which efficiently enhances conductivity and provides a large specific surface area for immobilizing mDNA-Js probes. The mDNA-J-assisted DNAzyme activated by Na^+^ binding hybridization chain reaction (HCR) promotes effective signal amplification, and owing to the specific recognition ability of ZrGA and mDNA-Js, the developed biosensor can accurately detect tumor-derived sEV-miRNA with high sensitivity and selectivity. Combined with a finger-sized U-disk wireless electrochemical analyzer (WEA) (plug and play), the proposed bioplatform is portable and relatively cheap (costs below $2 per test), making it promising for applications in areas with limited resources.

## Results and discussion

### Design of SPCE/ZrGA/mDNA-J bioplatform

**Fig. 1:**
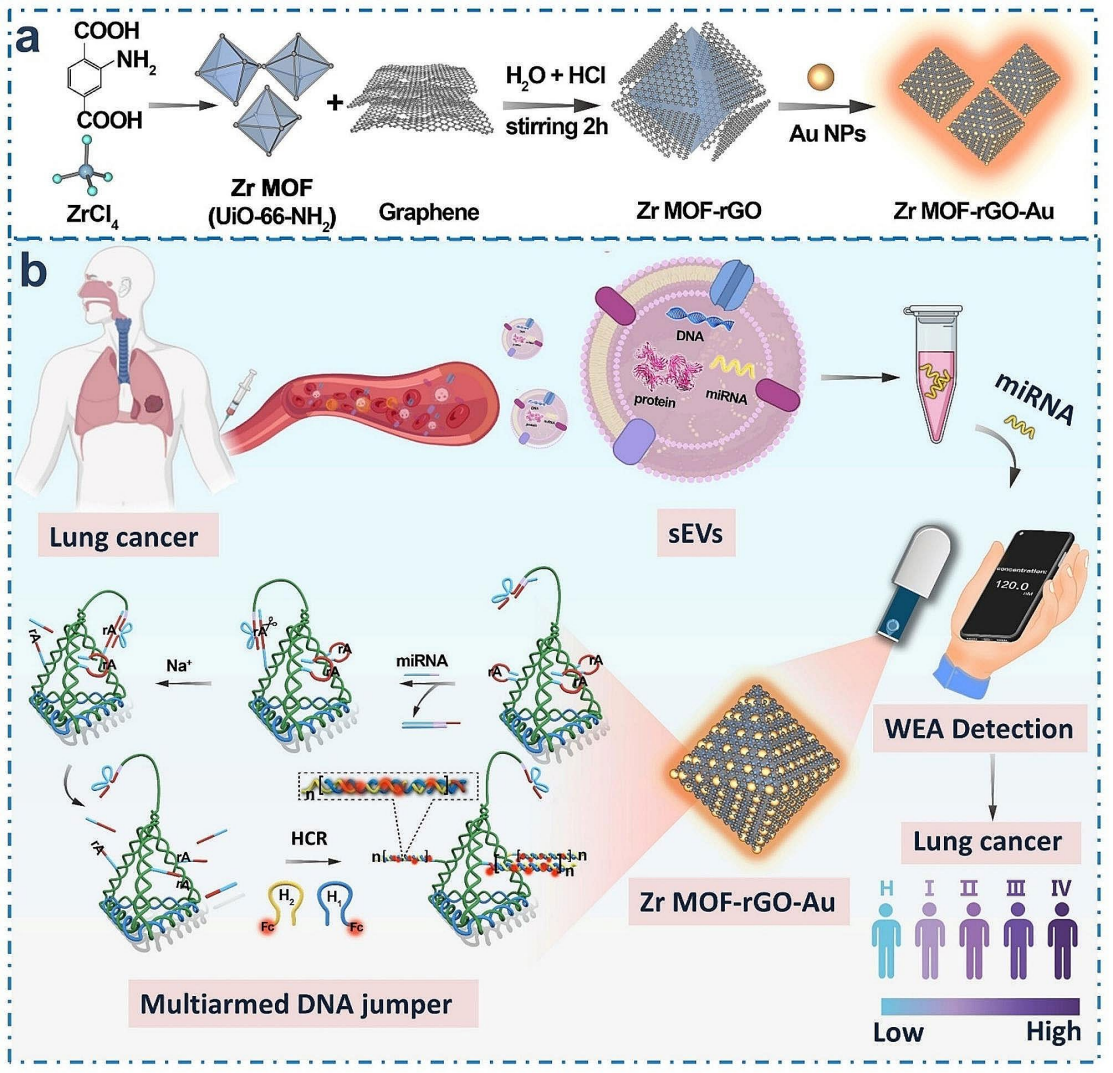
**(a)** Assembly of Zr-MOF-rGO-Au (ZrGA) and **(b)** schematic of small extracellular vesicle–derived microRNA (sEV-miRNAs) analysis for lung cancer diagnosis

Figure 1 shows the assembly of the Zr-MOF-rGO-Au (ZrGA) (Fig. 1a), the isolation and extraction of human blood sEV-miRNAs and the detection mechanism of sEV-miRNAs using the proposed portable SPCE/ZrGA/mDNA-J bioplatform (Fig. 1b). Combined with the finger-sized U-disk WEA (plug and play), reliable 2.4-G data transmission and up to 20-m transmission distance can be achieved (Figure S1). We prepared GO tightly packed Zr-MOF (Zr-MOF-rGO) via a one-step method, and the nanocomplex was modified on the SPCE surface. The synthesized graphene contacts all the faces of the MOF octahedron, which effectively accelerates the space charge separation and inhibits the recombination of electron–hole (*e*^*−*^*–h*^*+*^) pairs, affording accelerated interfacial electron transfer. After the electrodeposition of Au nanoparticles (NPs) to form SPCE/ZrGA, the DNA tetrahedron with three “robotic arms” (mDNA-J) was attached to SPCE/ZrGA by Au–S bonds.

As shown in the left enlarged part of Fig. 1b, mDNA-J comprises three pendulum-arm chains and a locus chain. The lower end of the pendulum-arm chains can closely bond with the three faces of the DNA tetrahedron and firmly attach to SPCE/ZrGA, and the upper end is the DNAzyme activated by Na^+^ and the LOCK chain domain. In the absence of target miRNA (miR-21, model), the substrate-cleaving ability of Na^+^-specific DNAzyme is locked owing to the hybridization between and their locking strands. However, in the presence of target miRNA, the locking strands sense and hybridize with the target miRNAs, release enzyme strands to open the hairpin structure, and then cleave their corresponding substrates, leaving a sticky end, which triggers HCR. The activated arm cuts the “rA” site on the lateral side of the mDNA-J (trace chain) skip by skip. The cut residues can be used as a “toehold” for HCR to form a stable dsDNA polymer in situ until the supply of H1 or H2 hairpins modified with “Fc” signal tags is exhausted. The redox signals from Fc are ultrasensitively measured to quantify and qualify the miRNA electrochemically owing to the excellent redox properties of Fc and the redox-signal-enhancing effect of SPCE/ZrGA/mDNA-J (detailed description, Figure S2).

### Characterization of SPCE/ZrGA bioplatform

**Fig. 2:**
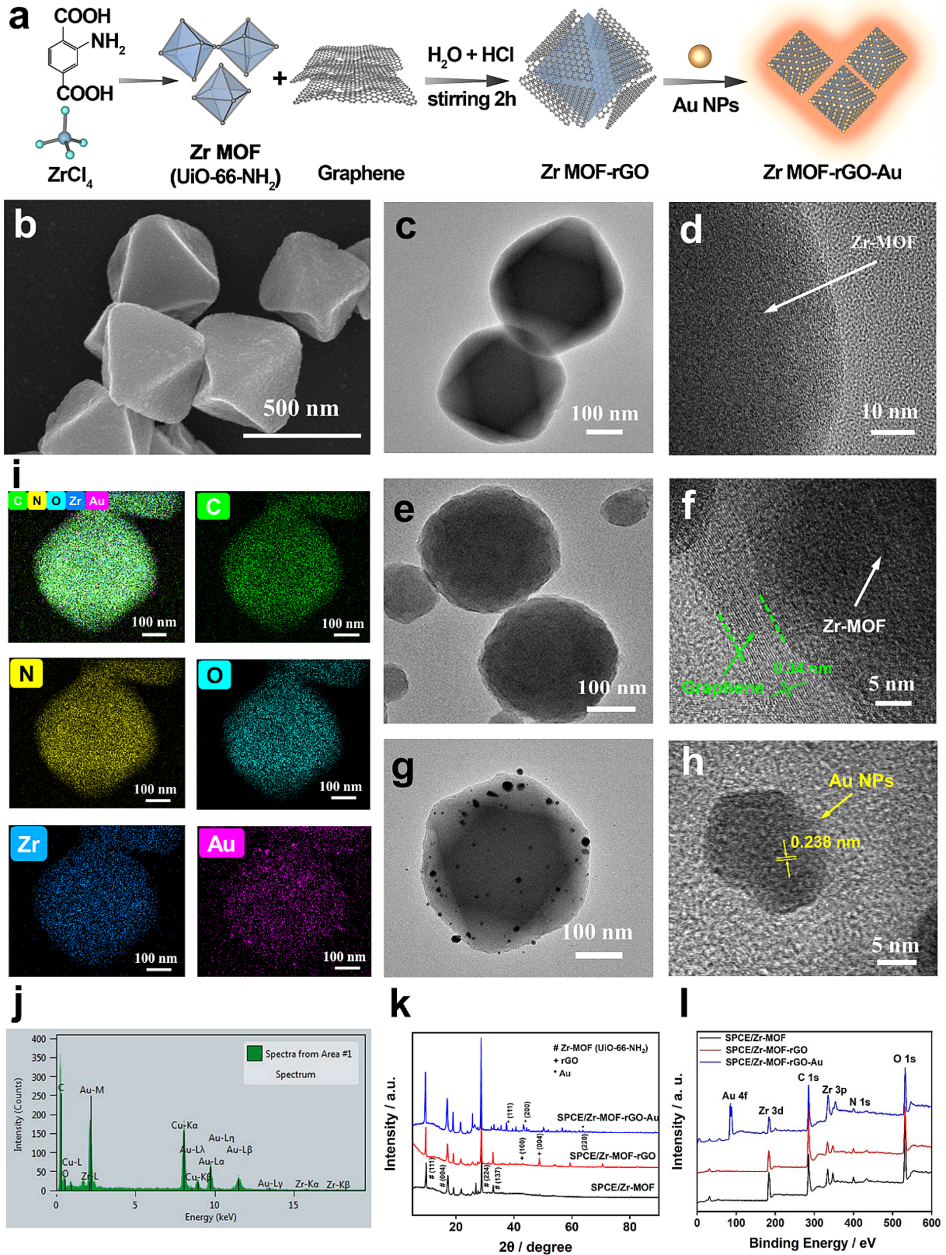
Characterization of the prepared electrodes: **(a)** assembly of Zr-MOF-rGO-Au (ZrGA). Transmission electron microscopy (TEM) images of **(b–d)** Zr-MOF, **(e-f)** Zr-MOF-rGO, and **(g–h)** ZrGA nanoparticles (NPs). **(i)** Energy-dispersive spectroscopy (EDS) elemental mapping and **(j)** spectrum of the elemental distribution of Zr-MOF-rGO-Au. **(k)** X-ray diffraction (XRD) and **(l)** X-ray photoelectron spectroscopy (XPS) images of Zr-MOF, Zr-MOF-rGO, and ZrGA

Figure 2a shows the assembly of the ZrGA. The particle size of Zr-MOF was observed using field-emission scanning electron microscopy (FE-SEM), which revealed that Zr-MOF has a uniform particle size (400 ± 50 nm) and a typical octahedral shape (Fig. 2b). Its morphology was further observed using transmission electron microscopy (TEM), and the obtained images are shown in Fig. 2c and d. Figure 2e and f show the morphologies of Zr-MOF-rGO with a graphene layer wrapping the Zr-MOF octahedron. High-resolution TEM (HR-TEM) revealed the edges of Zr-MOF and rGO (Fig. 2f), showing the lattice structure of rGO with a crystal plane spacing of 0.34 nm. Figure 2g and h show that Au NPs were successfully deposited on the Zr-MOF-rGO surface. The size of the Au NPs was 15 ± 5 nm, and the interplanar spacing was 0.238 nm, as revealed by HR-TEM (Fig. 2h). Further, energy-dispersive spectroscopy (EDS) showed that C, N, O, Zr, and Au were uniformly distributed on the ZrGA surface (Fig. 2i and j).

The XRD patterns of Zr-MOF (black), Zr-MOF-rGO (red) and ZrGA (blue) showed sharp diffraction peaks (Fig. 2k), which are consistent with the simulated data for the single crystal, indicating high purity and crystallinity [28, 42]. Figure 2l and S3a show full X-ray photoelectron spectroscopy (XPS) images of Zr-MOF, Zr-MOF-rGO, and ZrGA. Zr MOF-rGO-Au showed six characteristic peaks of O 1s, N 1s, C 1s, Zr 3p, Zr 3d, and Au 4 f. High-resolution XPS of C 1s for ZrGA showed a strong peak at the binding energies of 284.99 eV (Figure S3b), which is attributed to the sp^2^-hybridized C–C/C–H bond, and the peaks at 286.40 and 289.20 eV are attributed to the C–N and O–C = O bonds, respectively. The sample also showed two peaks ascribed to Zr 3d_3/2_ and Zr 3d_5/2_ (Figure S3c), and the peaks at the binding energies of 84.54 and 88.20 eV (Figure S3d) are attributed to the Au 4f_7/2_ and Au 4f_5/2_ chemical binding states of Au 4f, respectively, indicating that Au NPs were successfully synthesized on Zr-MOF-rGO.

### Characterization of the mDNA-J assembly

**Fig. 3:**
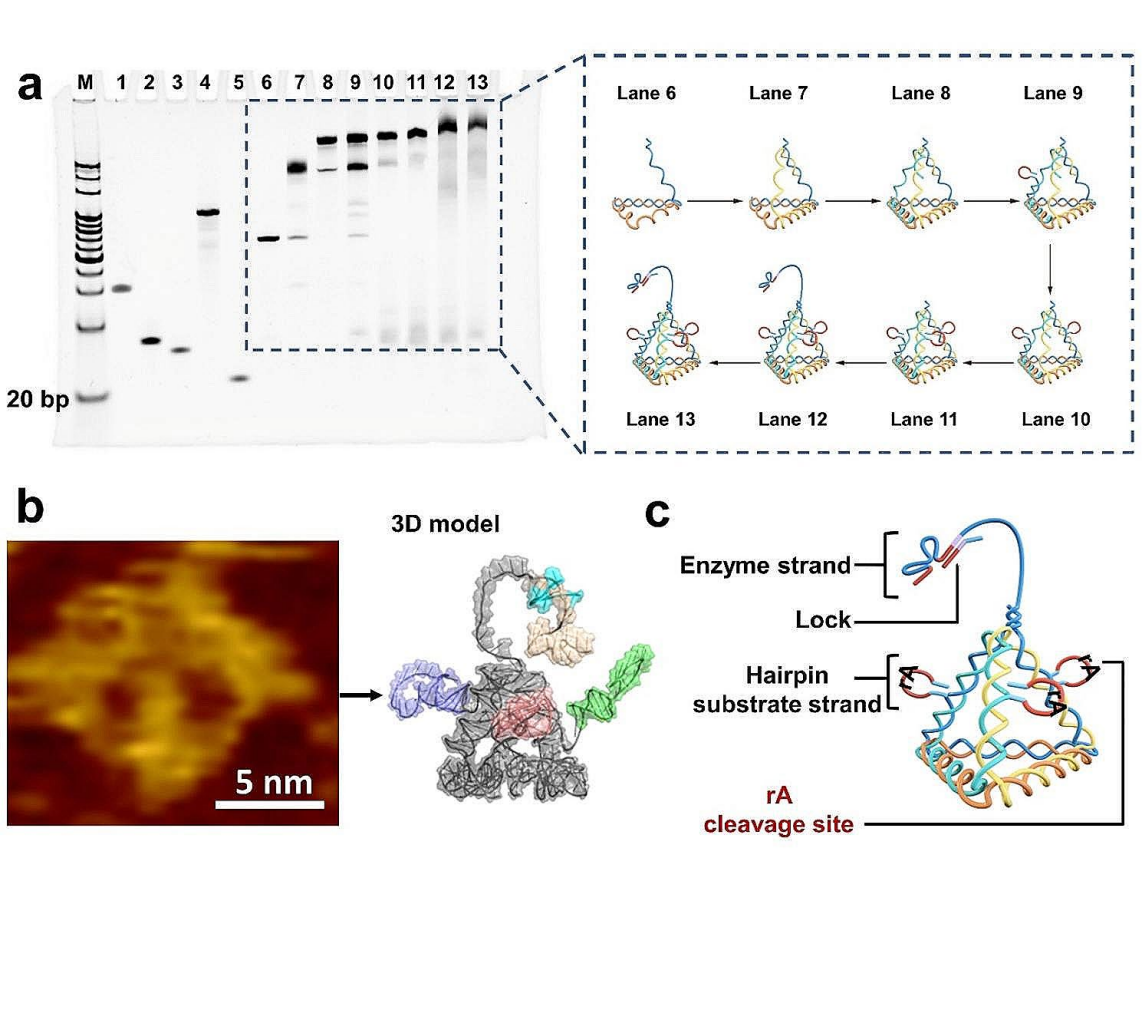
Characterization of the multiarmed DNA tetrahedral jumper (mDNA-J) assembly. **(a)** Polyacrylamide gel electrophoresis (PAGE) image of mDNA-Js. Lane M: 20 bp ladder; lane 1: S_4_; lane 2: S_1-a-SH_; lane 3: S_1-r-b_; lane 4: S_W_; lane 5: Lock; lane 6: S_4_ + S_1-a-SH_; lane 7: S_4_ + S_1-a-SH_ + S_2-a-SH_; lane 8: S_4_ + S_1-a-SH_ + S_2-a-SH_ + S_3-a-SH_; lane 9: S_4_ + S_1-a-SH_ + S_2-a-SH_ + S_3-a-SH_ + S_1-r-b_; lane 10: S_4_ + S_1-a-SH_ + S_2-a-SH_ + S_3-a-SH_ + S_1-r-b_ + S_2-r-b_; lane 11: S_4_ + S_1-a-SH_ + S_2-a-SH_ + S_3-a-SH_ + S_1-r-b_ + S_2-r-b_ + S_3-r-b_; lane 12: S_4_ + S_1-a-SH_ + S_2-a-SH_ + S_3-a-SH_ + S_1-r-b_ + S_2-r-b_ + S_3-r-b_ + S_W_; lane 13: S_4_ + S_1-a-SH_ + S_2-a-SH_ + S_3-a-SH_ + S_1-r-b_ + S_2-r-b_ + S_3-r-b_ + S_W_ + Lock (the detailed sequence is shown in Table S1 of the Supporting Information). **(b)** Atomic force microscopy (AFM) image of mDNA-Js and the corresponding 3D model. Scale bars, 5 nm. **(c)** Functional domains of the mDNA-Js

Native polyacrylamide gel electrophoresis (PAGE) and atomic force microscopy (AFM) were employed to evaluate the construction and reaction mechanism of the mDNA-J. As shown in lanes 1–13 (Fig. 3a, Table S1), with the addition of new strands, the migration distance decreased owing to the increase in molecular mass and the more complex spatial structure, and the Sw of a single chain showed a smaller migration distance owing to its long sequence (Fig. 3a, lane 4). mDNA-J migrated more slowly than other assemblies constructed by sequences of fewer than nine strands, and the clear bright band on the gel confirms the successful assembly and high yield of mDNA-Js (Fig. 3a, lane 13). AFM confirmed that the prepared mDNA-J has a tetrahedral structure with a diameter of ~ 5.8 nm (Fig. 3b).

As shown in Fig. 3c, the catalytic active core of the Na^+^-specific DNAzyme was separated into two halves by a locking strand (Lock), inhibiting its catalytic activity. The susceptibility of ribonucleotide to hydrolytic cleavage was 100,000-fold higher than that of its deoxyribonucleotide, a DNA-RNA chimeric sequence comprising an adenosine ribonucleotide (rA) flanked by two DNA domains of the hairpin substrate strand, which can hybridize to two arms of the enzyme strand. To avoid the steric interference of the DNA tetrahedron with the DNA hybridization and increase the accessibility of the hairpin substrate strand to the walking enzyme strand, we incorporated a poly-T spacer between the DNA tetrahedron and the enzyme strand. The DNA tetrahedrons could be anchored on the SPCE/ZrGA electrodes with a highly desirable orientation via Au–S chemical conjugation, and the unique pyramidal structure with three hairpin substrate strands shows that all immobilized hairpin substrate strands were distributed at fixed distances to the DNA tetrahedron, thereby maintaining spatial orientation for the effective assembly of the Na^+^-specific DNAzyme. The DNA tetrahedron was adopted as the foundation because a DNA tetrahedron of this size can be defined as a nanostructure, effectively decreasing the hindrance effect and maintaining spatial orientation for improved miRNA recognition.

### Electrochemical properties of SPCE/ZrGA/mDNA-J bioplatform

**Fig. 4:**
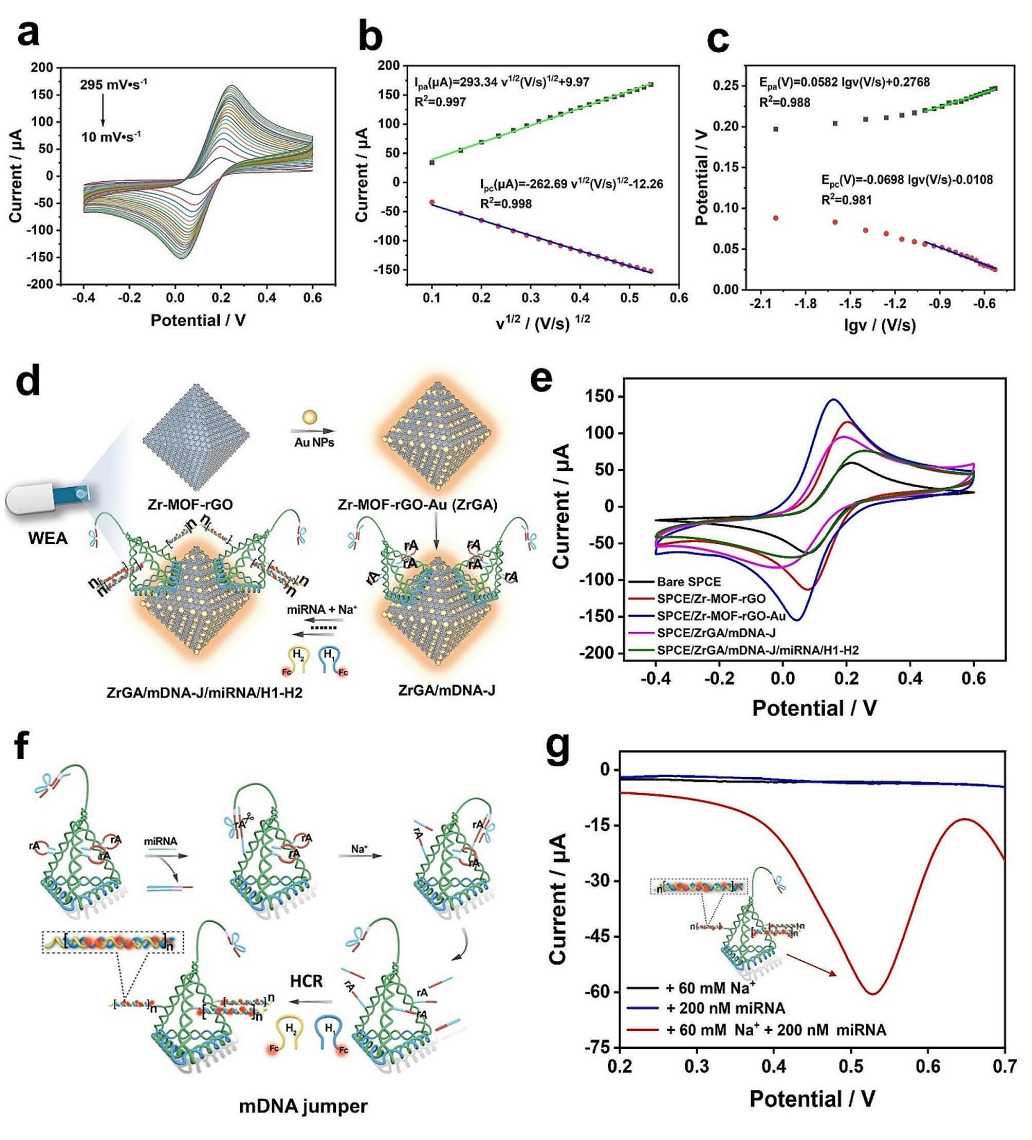
Electrochemical performance of the SPCE/ZrGA/mDNA-J bioplatform: **(a)** Cyclic voltammetry (CV) curves of SPCE/ZrGA at different scan rates in 5 mM [Fe (CN)_6_]^3−/4−^ containing 0.1 M KCl. **(b)** Variation of anodic and cathodic peak currents with scan rate. **(c)** Curve fitting of the logarithm of the scan rate (lgv) versus anode/cathode potential. **(d)** Flow diagram of miRNA detection by the proposed SPCE/ZrGA/mDNA-J bioplatform. **(e)** CV curves of bare SPCE, SPCE/Zr-MOF-rGO, SPCE/ZrGA, SPCE/ZrGA/mDNA-J, and SPCE/ZrGA/mDNA-J/miRNA/H1-H2 in 5 mM [Fe (CN)_6_]^3−/4−^ containing 0.1 M KCl. Feasibility study: **(f)** Flow diagram of miRNA detection by mDNA jumpers. **(g)** Square wave voltammetry (SWV) responses of the prepared electrode in the presence of 200-nM miR-21 and 60-mM Na^+^ (0.01 M PBS solution; scan rate: 50 mV s^− 1^)

The properties of the raw materials for the bioplatform are vital. Thus, cyclic voltammetry (CV) was employed to evaluate the electrochemical performance of the SPCE/ZrGA bioplatform at different scan rates (10–295 mV s^− 1^) in 5 mM [Fe(CN)_6_]^3−/4−^ containing 0.1 M KCl (Fig. 4a). Figure 4b shows the variation of peak currents with the square of the scan rate. Both the anode and cathode peak currents showed linear relationships with the equations I_*p.a.*_(µA) = 293.34v^1/2^(V s^− 1^)^1/2^ +9.97 (R^2^ = 0.997) and I_*pc*_(µA) = − 262.69v^1/2^(V s^− 1^)^1/2^-12.26 (R^2^ = 0.998), respectively. This oxidation–reduction reaction of SPCE/ZrGA indicates a diffusion-controlled process. Furthermore, the electroactive surface area (A) of the three electrodes was calculated using the *Randles–Sevcik* equation: [28]

1 $${I}_{\text{p}}=2.69\times {10}^{5}{n}^{\frac{3}{2}}A{D}^{\frac{1}{2}}{\nu }^{\frac{1}{2}}{C}_{0}$$

where *I*_p_ is the peak current (A), n (= 1) is the number of electronic transfers, *D* (= 6.7 ± 0.02 × 10^–6^ cm^2^ s^− 1^) is the diffusion coefficient, *υ* = 0.05 V s^− 1^, and *C*_*0*_ (= 5 × 10^− 6^ mol cm^–3^) is the [Fe(CN)_6_]^3−/4−^concentration. According to Eq. (1), SPCE/ZrGA has an electroactive surface area of 0.191 cm^2^, which is 1.3 times that of SPCE/Zr-MOF-rGO (0.148 cm^2^) and 2.5 times that of SPCE (0.076 cm^2^). In addition, Fig. 4c shows the variation of the anode and cathode peak potentials with the logarithm of the scan rate (lg(v)). The equation of line for the anode peak potential is E_*p.a.*_(V) = 0.0582 lgv (V s^− 1^) + 0.277 (R^2^ = 0.988), and that of the cathode is E_*pc*_(V) = -0.0698 lgv (V s^− 1^) + 0.011 (R^2^ = 0.981). Based on the Laviron theory: [44]

2 $$lg\frac{{k}_{a}}{{k}_{c}}=lg\frac{\alpha }{1-\alpha },$$

3 $$\begin{gathered} lg{k_s} = \alpha \,{\text{lg}}\left( {1 - \alpha } \right) + \left( {1 - \alpha } \right)l\,g\alpha - l\,g \\ \frac{{RT}}{{nF\nu }} - \frac{{2.3RT\alpha }}{{\left( {1 - \alpha } \right)nF\vartriangle {E_p}}}, \\ \end{gathered}$$

where *k*_*a*_ and *k*_*c*_ are the slopes of *E*_*p.a.*_*–lg(v)* and *E*_*pc*_*–lg(v)* lines, respectively, *R* is the gas constant (8.314 J·(mol·K) ^−1^), *T* is the absolute temperature (298 k), *F* is the Faraday’s constant (96,493 C·mol^− 1^), and n is the number of electronic transfers (= 1). From Eqs. (2) and (3), *α* = 0.455, which is the charge transfer coefficient, and *k*_*s*_ = 1.243 s^− 1^, which is the apparent electron transfer rate constant. The electron transfer rate *k*_*s*_ obtained here is higher or comparable to the values reported in the literature (Table S3), indicating that ZrGA exhibits accelerated electron transfer.

Figure 4d shows the assembly process of the SPCE/ZrGA/mDNA-J bioplatform, and CV was employed to characterize the electrochemical behavior of the electrode during its modification on WEA. The CV curves of different modified electrodes in a 5 mM [Fe (CN)_6_]^3−/4−^ detection solution containing 0.1 M KCl are shown in Fig. 4e. After the Zr-MOF-rGO nanocomposite was modified on the surface of the SPCE electrode (red curve), the current response was significantly higher than that of the bare SPCE electrode (black curve). This is because, in addition to the good conductivity of the Zr-MOF-rGO nanocomposite, it greatly increased the specific surface area of the electrode. Notably, the current signal response of ZrGA (blue curve) was approximately three times that of Zr-MOF-rGO, indicating that Zr-MOF-rGO provided a large specific surface area to support Au NPs and could immobilize numerous capture probes. When the tentacles of mDNA-Js were fixed on the electrode surface by Au–S bonds (SPCE/ZrGA/mDNA-J, purple curve), the current response significantly decreased. This is attributed to the electrostatic repulsion between the self-negatively-charged phosphate skeleton and Fe^2+/3+^ in the solution, which hindered current diffusion between [Fe(CN)_6_]^3−/4−^ and the electrode surface, thereby decreasing the redox peak current. Due to the hybridization and polymerization of more non-electroactive DNA chains, the current response further decreased after HCR (SPCE/ZrGA/mDNA-J/miRNA/H1-H2, green curve). This is because the DNA hybridization double chains formed on the electrode surface further hinder current diffusion from [Fe(CN)_6_]^3−/4−^ to the electrode surface, thereby decreasing the current response signal.

A feasibility study was conducted by measuring the change in the target miRNA-induced electrochemical signal in the presence of 200 nM miR-21 (target) and 60 mM Na^+^ in a 0.01 M PBS solution. The detection procedure is depicted in Fig. 4f g. miR-21 was selected herein to demonstrate the performance of the proposed platform because it is a prevalent circulating miRNA biomarker overexpressed in lung cancer. The target miR-21 serves as a “toehold” to initiate interactions with the blue domain of Lock. Subsequently, the complementary double chain (△*G*_enzyme strand: Lock_ = − 26.62 kcal mol^− 1^) formed partly by enzyme strands and partly by Lock is opened and forms an miR-21–Lock complex (△*G*_miR-21: Lock_ = − 33.35 kcal mol^− 1^). Thus, the red domain of the enzyme strand is no longer occluded and can bind to the red domain of the hairpin substrate strand (△*G*_substrate strand_ = − 5.76 kcal mol^− 1^), then opens the hairpin substrate strand and forms a Na^+^-specific DNAzyme (△*G*_enzyme strand: substrate strand_ = − 34.06 kcal mol^− 1^). With the addition of Na^+^, DNAzyme is activated to specially cut the substrate strand. The leaving sticky end opens hairpin H1 (△*G*_H1_ = − 3.75 kcal mol^− 1^) and forms a sticky-end–H1 double chain (△*G*_sticky end: H1_ = − 25.10 kcal mol^− 1^), at which a new sticky end opens hairpin H2 (△*G*_H2_ = − 6.19 kcal mol^− 1^) and forms a sticky-end–H1–H2 double chain (△*G*_sticky end: H1:H2_ = − 43.97 kcal mol^− 1^). The constant existence of sticky ends induces HCR (H1 and H2 structures, Figure S4).

Figure 4 g shows square wave voltammetry (SWV) curves of the SPCE/ZrGA/mDNA-J bioplatform under different detection conditions. There was almost no current response for the SPCE/ZrGA/mDNA-J bioplatform in the presence of only 60 mM Na^+^ (black curve) or miR-21 (target, blue curve), indicating that HCR was not induced. A significant SWV response peak (Fc, 0.52 V) was observed in the presence of miR-21 and Na^+^ (red curve), indicating that HCR was induced and DNA hairpins with Fc (H1-Fc and H2-Fc) were opened to hybridize with each other.

### Detection of sEV-miR-21

Optimization of conditions: To further optimize the SPCE/ZrGA bioplatform, we first optimized the mDNA-J concentration and incubation time immobilized on the sensing surface of SPCE/ZrGA. When a parameter is optimized, other parameters would be optimal. The detection sensitivity of the SPCE/ZrGA bioplatform for biomolecules largely depends on the number of fixed mDNA-Js. Figure S5 shows that the SWV current signal detected by 0.2 µM miR-21 at an mDNA-J concentration range of 0.2–1.2 µM rapidly increased at the beginning and reached a maximum at 1.0 µM and then remained constant. As the incubation time increased from 30 to 150 min, the generated current signal increased almost linearly within the first 120 min and then remained constant afterward (Figure S6). In addition, the time for H1–H2 HCR contributed considerably to the total detection time (Figure S7). In the time range of 30–150 min, the SWV response signal increased almost linearly within the first 60 min and then stabilized, indicating that the reaction reached a steady state. Therefore, for an mDNA-J concentration of 1.0 µM, an mDNA-J incubation time of 150 min and an HCR time of 60 min were selected for subsequent experiments.

**Fig. 5:**
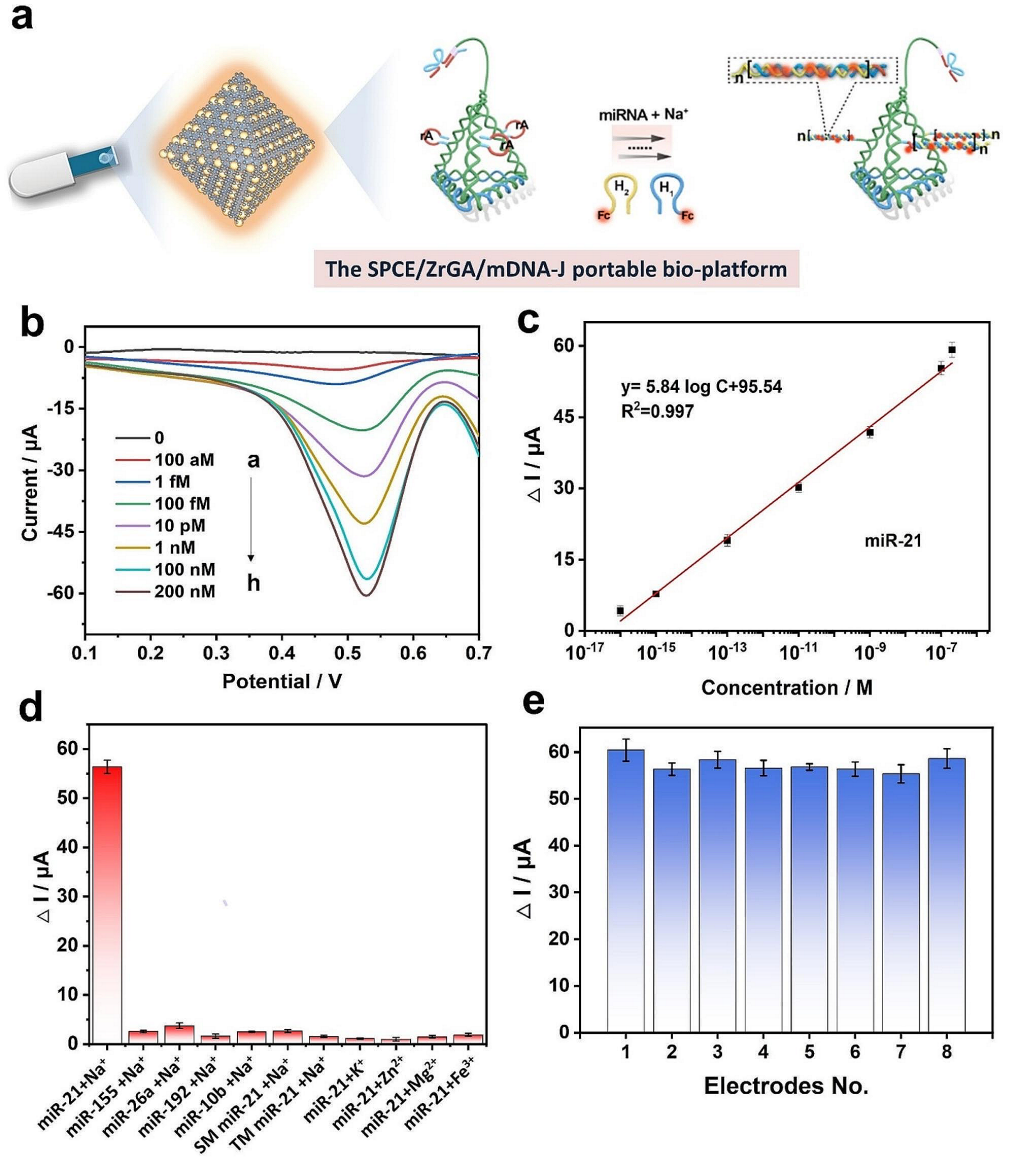
**(a)** Schematic of the SPCE/ZrGA/mDNA-J bioplatform for the detection of miR-21. **(b)** SWV responses to different concentrations of miR-21: (a) 0 M, (b) 100 aM, (c) 1 fM, (d) 100 fM, (e) 10 pM, (f) 1 nM, (g) 100 nM, and (h) 200 nM. **(c)** Calibration plots of **(b). (d)** Current responses of the SPCE/ZrGA/mDNA-J electrode with the target (miR-21), interfering miRNAs (miR-155, miR-26a, miR-192, miR-10b, single-base mismatch (SM) miR-21, three-base mismatch (TM) miR-21), and different cofactors (K^+^, Zn^2+^, Mg^2+^, and Fe^3+^). Error bars: SD; *n* = 3. **(e)** Reproducibility of the proposed SPCE/ZrGA/mDNA-J biosensor (*n* = 8). The error bars indicate standard deviations for five measurements

Figure 5a shows the response of the SPCE/ZrGA/mDNA-J bioplatform for the detection of miR-21 in the presence of 200 nM miR-21 (target) and 60 mM Na^+^. Under optimal experimental conditions, the current responses of the SPCE/ZrGA/mDNA-J bioplatform to the target miR-21 at various concentrations (0–0.2 µM) were examined using the SWV method (Fig. 5b). With an increase in the concentration of the target miR-21, the Fc signals gradually increased. The relationship between the concentration and the current signal was fitted to a linear function (Fig. 5c). For the concentration range of 100 aM–0.2 µM, the equation for the linear fitting is y = 5.84 logC _miR-21_ + 95.54 (R^2^ = 0.997), where C is the concentration of the targeted miR-21. The calculated LOD for miR-21 is 34.6 aM (S/*N* = 3). Considering the detection range and LOD, the performance of the SPCE/ZrGA/mDNA-J bioplatform is comparable to or better than that of previously reported biosensors (Table S4). Notably, this bioplatform is promising for POC applications because it does not require target amplification, making it less time-consuming and easy to operate (it can be operated by merely dropping 10 µL of a reactive solution).

### Specificity and reproducibility of the proposed SPCE/ZrGA/mDNA-J bioplatform

To further investigate the selectivity of the SPCE/ZrGA/mDNA-J bioplatform, we introduced a variety of control targets including mismatched targets based on binding free energy changes via the NUPACK and different cofactors (K^+^, Zn^2+^, Mg^2+^, and Fe^3+^) to conduct anti-interference experiments. As shown in Fig. 5d, the peak current change (△I) was highest after the hybridization of the complementary target (miR-21) with 60 mM Na^+^, whereas other interfering miRNAs and different cofactors produced very weak current signals. This is attributed to the high specificity of chain substitution reactions. The reproducibility of the electrode was further studied (Fig. 5e). We measured the SWV current response of 0.2 µM miR-21 in eight ZrGA biosensors, and the calculated relative standard deviation is 2.49% (miR-21), indicating good repeatability.

### sEV-miR-21 clinical sample detection and comparison

**Fig. 6:**
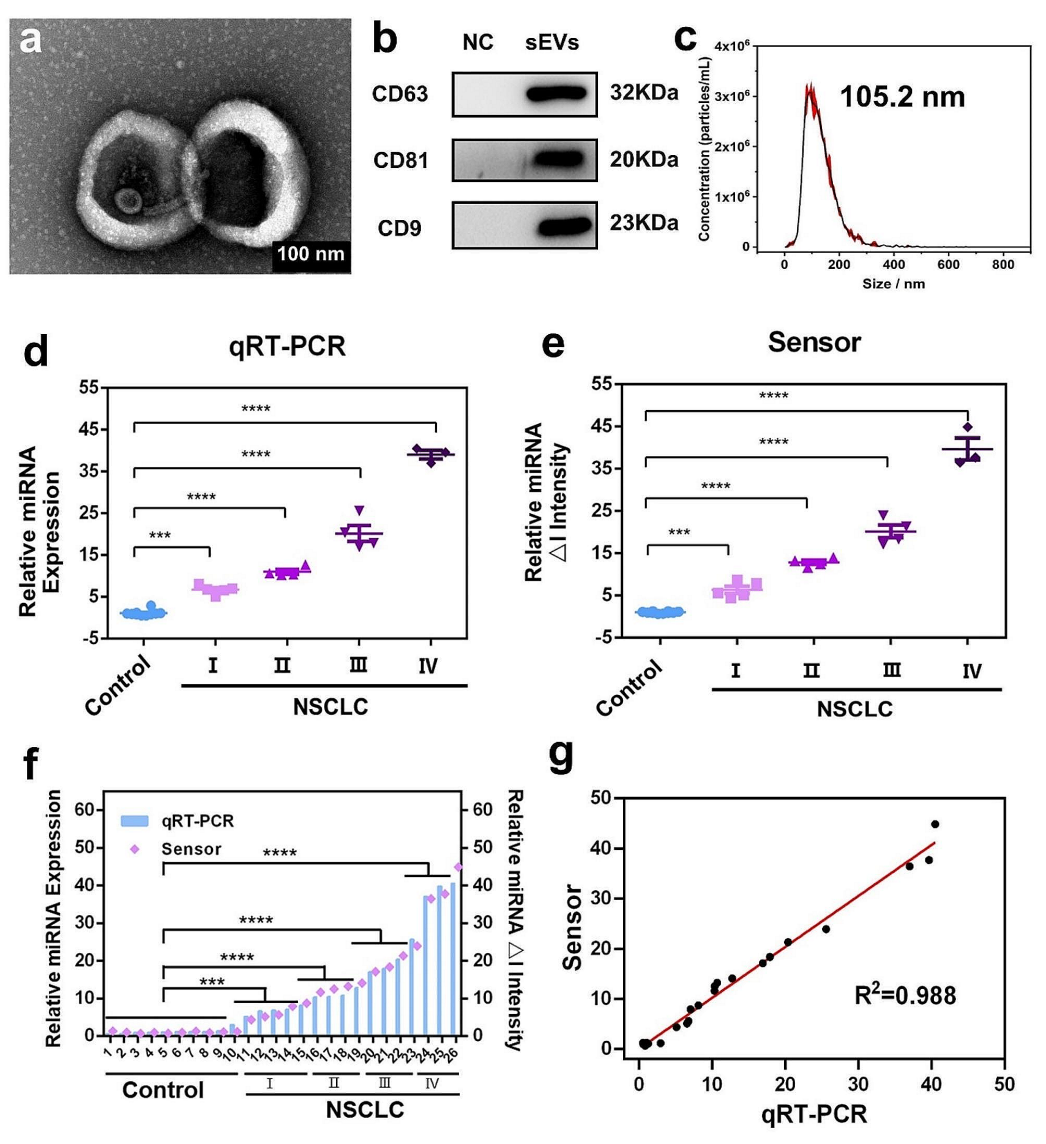
**(a)** TEM image of isolated sEVs derived from clinical blood samples. The scale bar is 100 nm. **(b)** Western blot bands of CD63, CD81, and CD9 on the sEV membrane, NC: PBS. **(c)** Nanoparticle tracking analysis (NTA) result of sEVs. **(d)** and **(e)** Validation of clinical differentiation for sEVs miR-21 in clinical samples from healthy individuals (10 samples as a control) and non-small-cell lung cancer (NSCLC) patients (16 samples) who were in the tumor stage using qRT-PCR and our platform, respectively. The results were analyzed by an unpaired, two-tailed Student’s t-test (two groups) or ANOVA (three or more groups) followed by Bonferroni’s correction if needed. ***: *p* < 0.001, ****: *p* < 0.0001. **(f)** Comparison between our platform and qRT-PCR towards sEVs miR-21 detection. **(g)** Correlation between the results of sEVs miR-21 detection measured using the proposed sensor and qRT-PCR

The morphology, characteristic proteins, and particle-size distribution of the extracted sEVs were analyzed. TEM revealed that the sEVs have a cup-shaped membrane structure (Fig. 6a and S8), which is consistent with previous reports [48, 49]. For the protein expression, western blot (WB) experiments confirmed the presence of characteristic protein markers, CD63, CD81, and CD9, on the sEVs membrane (Fig. 6b), corresponding to the 32, 20, and 23 kDa bands, respectively, which is consistent with previous reports. Furthermore, NP tracking analysis (NTA) revealed that about 98% of the sEVs from serum specimens have a particle size of 30–250 nm with a mean of 105.2 nm (Fig. 6c). Therefore, the extracted sEVs maintained a good membrane structure and significant distribution of specific proteins, demonstrating the effective extraction of sEVs from serum specimens.

To confirm the applicability of the proposed SPCE/ZrGA/mDNA-J portable bioplatform to clinical samples, we explored its response to sEV-miR-21 in total RNA extracted from sEVs of clinical plasma samples. We analyzed 26 clinical blood samples (10 samples from healthy individuals and 16 samples from non-small-cell lung cancer (NSCLC) patients) using the proposed bioplatform and quantitative real-time PCR (qRT-PCR), and both techniques showed similar results (Fig. 6d and e). The signal of the proposed SPCE/ZrGA/mDNA-J bioplatform in the clinical sample tests increased proportionally with NSCLC staging in the samples collected from NSCLC patients. Furthermore, a much higher expression of miR-21 was observed in stage IV patients compared with that of healthy individuals, which is consistent with previous reports [28, 50]. Moreover, the proposed bioplatform and classic qRT-PCR showed similar results in differentiating samples from NSCLC patients (Fig. 6f), indicating good consistency between the proposed bioplatform and qRT-PCR (R^2^ = 0.988) (Fig. 6g). These results show that the proposed bioplatform has high accuracy and applicability and can accurately reflect NSCLC staging in clinical samples.

## Conclusions

Herein, we developed a SPCE/ZrGA/mDNA-J bioplatform for detecting trace miRNA by combining the ZrGA nanocomplex and mDNA-J assisted DNAzyme activated by Na^+^. The developed portable biosensor can detect miR-21 with high sensitivity (LOD as low as 34.6 aM), which is better than that of conventional techniques. Thus, the proposed biosensor is promising for POC applications. Furthermore, the biosensor can detect mutations, which is important worldwide. Additionally, the signal of the biosensor increases proportionally with NSCLC staging in clinical samples. The proposed bioplatform and classical qRT-PCR showed similar results (R^2^ = 0.988) in distinguishing NSCLC samples.

The exceptional sensing performance of the proposed SPCE/ZrGA/mDNA-J bioplatform is attributed mainly to the synergistic effects of the following. (1) The tightly graphene-wrapped Zr-MOF octahedral complex accelerates space charge separation and inhibits photogenic *e*^*−*^*–h*^*+*^ pair recombination, affording an ultrahigh conductivity of the bioplatform and a large surface area of ZrGA, providing numerous fixed sites for mDNA-J and increasing the sensitivity; (2) the precise and controllable 3D nanostructure and multiarm structure of mDNA-J probes ensure the rigidity and orientation of the probe array at the sensing interface, which aids precise identification units and enhances the selectivity and detection efficiency; (3) the ZrGA-modified paper-based biosensor was fabricated along with a commercial SPE and a wireless USB-type electrochemical device (plug and play) that generates local molecular constraints through the 3D nanostructure, increasing the collision probability of trace target molecules in a microreaction system (~ 10 µL) and making the sensor suitable and sensitive for POC diagnosis in areas with limited resources. In summary, the proposed electrochemical biosensor is promising for monitoring diverse tumor biomarkers in POC biosensing through a simple, accuracy, low-cost (costs below $2 per test) and less time-demanding approach.

### Electronic supplementary material

Below is the link to the electronic supplementary material.

Supplementary Material 1

## Data Availability

The data that support the ffndings of this study are available in the supplementary material of this article.

## Declarations

### Supporting information

Chemical and instruments, experimental procedures, oligonucleotide sequences, and supplementary figures are available is available from the Journal of Nanobiotechnology Online Library or the author.

## Abbreviations

AFM: Atomic force microscopy
CV: Cyclic voltammetry
EDS: Energy-dispersive spectroscopy
GO: Graphene oxide
HCR: Hybridization chain reaction
LOD: Limit of detection
MOF: Metal–organic frameworks
mDNA-J: Multiarmed DNA tetrahedral jumper
NSCLC: Non-small-cell lung cancer
NTA: Nanoparticle tracking analysis
POC: Point-of-care
SPCE: Screen-printed carbon electrode
TEM: Transmission electron microscopy
sEV: Small extracellular vesicle
SWV: Square wave voltammetry
SM: Single-base mismatch
TM: Three-base mismatch
WB: Western blot
WEA: Wireless electrochemical analyzer
XPS: X-ray photoelectron spectroscopy
XRD: X-ray diffraction
ZrGA: Zr-MOF-rGO-Au
