# Supplementary material for: Multiarmed DNA jumper and metal-organic frameworks–functionalized paper-based bioplatform for small extracellular vesicle–derived miRNAs assay
Source: J Nanobiotechnology. 2024 May 22;22:274. doi: 10.1186/s12951-024-02546-w (PMC11110235; doi:10.1186/s12951-024-02546-w)
Supplement: Supplementary file 1 — Supplementary Material 1 [file 12951_2024_2546_MOESM1_ESM.docx]

**Supporting Information**

**Multiarmed DNA jumper and Metal-Organic Frameworks–Functionalized Paper-based Bioplatform for Small Extracellular Vesicle–derived miRNAs Assay**

Xiaopei Qiu^a,1^, Huisi Yang^b,1^, Man Shen^a^, Hanqing Xu^a^, Yingran Wang^a^, Shuai Liu^a^, Qian Liu^a^, Minghui Sun^a^, Zishan Ding^a^, Ligai Zhang^a^, Jun Wang^a^, Taotao Liang^c^, Dan Luo^d^, Mingxuan Gao^a,^*, Ming Chen ^a,e,^*, and Jing Bao^a,^*

^a^ Department of Clinical Laboratory Medicine, Southwest Hospital, Third Military Medical University (Army Medical University), Chongqing, 400038 PR China

^b^ Key Laboratory for Biorheological Science and Technology of Ministry of Education, State and Local Joint Engineering Laboratory for Vascular Implants, Bioengineering College of Chongqing University, Chongqing 400044, PR China

^c^ Chongqing Sports Medicine Center, Department of Orthopedic Surgery, Department of Clinical Laboratory Medicine, Southwest Hospital, the Third Military Medical University, Chongqing 40038, P.R. China

^d^ Department of Biological and Environmental Engineering, Cornell University, Ithaca, NY 14853-5701, USA

^e^ College of Pharmacy and Laboratory Medicine, Army Medical University, 30 Gaotanyan, Shapingba District, Chongqing 400038 PR China

^1^ These authors contributed equally to this work.

* Corresponding author. E-mail addresses: mingxuan_gao@163.com (M. Gao), chming1971@126.com (M. Chen), baojing_1991@163.com (J. Bao).

**CONTENT**

EXPERIMENTAL SECTION 3

*Chemical and materials* 3

*Apparatus and electrochemical measurements* 3

*Preparation of Zr-MOF-rGO* 4

*Fabrication of the SPCE/Zr MOF-rGO-Au bioplatform* 5

*Self-assembly of mDNA-Js* 5

*Detection of sEV-miR-21* 5

*Clinical sample detection and methodology comparison* 6

FIGURES 8

TABLES 16

REFERENCES 20

# Experimental section

**Chemical and materials**

Oligonucleotides were synthesized and purified by Sangon Biotechnology Co. Ltd. (Shanghai, China). Buffer solutions were purchased from Sangon Biotechnology Co. (Shanghai, China), and Super GelRed nuclear staining was obtained from US Everbright Inc. (China). The RNase inhibitor and 20 bp DNA ladder were obtained from TaKaRa Biotechnology Company (Dalian, China). Zirconium oxide chloride octahydrate (ZrCl_4_), hydrogen tetrachloroaurate trihydrate (HAuCl_4_•4H_2_O), tris-(2-carboxyethyl) phosphine hydrochloride (TCEP), and 6-mercapto-1-hexanol (MCH) were purchased from Aladdin Reagent Co., Ltd (Shanghai, China). Certified fetal bovine serum was purchased from VivaCell (Shanghai, China), and tris-HCl was obtained from Sigma Aldrich and used as received (St. Louis, USA). The SPCE was purchased from Zensor R&D Co (Taiwan, China). All reagents were of analytical reagent grade and used without further treatment. All experiments involving miRNA were conducted in an RNase-free environment. All buffer solutions were treated with 0.1% diethyl pyrocarbonate and autoclaved, and the base sequences are summarized in Tables S1 and S2.

**Apparatus and electrochemical measurements**

The morphologies and sizes of the as-prepared electrodes were characterized using FE-SEM (FEI Nova 400) and TEM (Talos F200X). XRD was performed using Bruker AXS D5005, and XPS was conducted using Escalab 250Xi instrument (Thermo, USA). AFM was performed using Asylum Research Cypher. The size distribution of sEVs was analyzed by NTA (Nanosight NS300, UK). RT-qPCR was performed using the Bio-Rad CFX96 thermal cycler (Bio-Rad, USA), which was also used to analyze the reaction products. The PAGE images were captured using Bio-Rad ChemDoc XRS (Bio-Rad, USA). All electrochemical experiments were performed using a CHI 760E electrochemical workstation (Shanghai Chenhua Instruments Co. Ltd, China) and a WEA (ECWP100C, Zensor R&D Co, China.)

**Preparation of Zr-MOF-rGO**

In a typical synthesis with some modifications,^1^ ZrCl_4_ (0.233 g), and 2-aminoterephthalic acid (BDC-NH_2_) (0.181 g) were first dissolved in 102.1 mL N,N-dimethylformamide (DMF). Then, acetic acid (22.9 mL) was added to the solution, followed by 20-min ultrasonication. The solution was then transferred to a 200-mL Teflon-lined stainless steel autoclave and heated at 120 ℃ for 16 h. After cooling to room temperature, the products were collected via centrifugation and washed three times with DMF to remove the excess reactants. Thereafter, they were washed three times with methanol to remove DMF and finally heated at 150 ℃ in a vacuum for 12 h. For 0.1-g Zr-MOF octahedrons, 30 mL of deionized water and 1 mM HCl were mixed in a glass vial and ultrasonicated for 10 min. Thereafter, 0.05 g of GO powder was added to the system, and the system was ultrasonicated for 10 min and stirred for 2 h. Then, the products were collected via centrifugation at 5000 rpm and dried in air at 80 ℃.

**Fabrication of the SPCE/Zr MOF-rGO-Au bioplatform**

The dried Zr-MOF-rGO (2.0 mg) nanocomposites were dispersed in 1 mL of primary water and sonicated for 2 h to obtain a uniformly dispersed solution. The SPCE has a complete three-electrode system, which can be used without pretreatment. Further, 10 μL of the mixed solution was added dropwise to the prepared clean SPCE electrode and dried in air at room temperature to obtain different modified electrodes. The prepared electrodes were washed with deionized water. Then, the Zr-MOF-rGO complex was obtained by depositing a mixed solution of 0.01 M Na_2_SO_4_ and H_2_SO_4_ containing 5 mM HAuCl_4_ for 200 s at −0.2 V (vs. ref. electrode) through amperometry chronoamperometry (i–t) technique (SPCE/ZrGA).

**Self-assembly of mDNA-Js**

Nine ssDNAs were hybridized to form mDNA-J (Table S1). The nine ssDNAs were mixed in equal proportions in the TCEP (3 mm)-containing TM buffer solution (20 mM Tris, 50 mM MgCl_2_6H_2_O, pH 8.0) to prepare DNA nanostructures with a final concentration of 0.8 µM. Then, the solution was heated to 95 ℃ and held for 10 min, after which it was gradually cooled to 4 ℃ and maintained for more than 10 min, and mDNA-J was formed. The formation of the DNA nanostructure was analyzed using 8% PAGE in a TBE buffer at 80 and 110 V for 10 and 40 min, respectively.

**Detection of** **sEV-miR-21**

To fabricate the SPCE/ZrGA bioplatform, the electrodes were immersed in a 20-μL 1 μM mixed solution of mDNA-Js, incubated at 37 ℃ for 2 h, and then rinsed three times with 0.01 M PBS. After that, an MCH solution (1.0 mM) was used for sealing treatment (15 min) to remove nonspecific effects. Then, the prepared electrode in a hybrid mixture containing miR-21 with different concentrations as well as H1-Fc and H2-Fc, and initiating a series of strand displacement reactions. The hybridization reaction was performed at 37 ℃, rinsed three times with 0.01 M PBS, and recorded by SWV. The LOD was determined by finding the concentration at which the signal, after background subtraction, was three times higher than the standard deviation for the concentration (S/N = 3).

**Clinical sample detection and methodology comparison**

In total, 26 clinical blood samples, including 16 samples from NSCLC patients and 10 samples from healthy individuals (as a control), were analyzed using the proposed SPCE/ZrGA bioplatform and classic qRT-PCR. This study was approved by the Ethics Board, and all human blood samples were obtained from the Southwest Hospital (Chongqing). The serum was centrifuged at 3000 rpm for 10 min to remove blood cells. Next, the supernatant was centrifuged at 1600×g for 10 min to remove cell debris, and the supernatant was centrifuged at 10000×g for 10 min to remove large vesicles. The media supernatant was passed through a 0.22-μm pore filter (Millipore). Next, sEVs were obtained using an exoRNeasy Midi/Maxi Kit. The obtained sEVs were resuspended in precooled PBS and stored at −80 ℃ until time for use. The concentration and size distribution of sEVs were analyzed via NTA, which were performed on the Zetasizer Nano ZS90 and followed by protocol. Western blotting was utilized to evaluate the distinctive proteins of sEVs (CD63, CD81, and CD9) according to a predetermined methodology. In brief, the cells and sEVs were lysed with RIPA buffer on ice and their protein content was determined by BCA method. The proteins were then separated by SDS-PAGE electrophoresis. The separated proteins were transferred to a PVDF membrane, blocked with 5% nonfat milk for 1 hour, and then incubated with specific primary antibodies overnight at 4°C. After washing, the membrane was incubated with a secondary antibody conjugated for 1 hour at room temperature. Finally, the protein expressions were measured using a ChemiDocTM MP imaging system.

To extract sEV-derived total RNAs, first, we added 600 μL of Trizol reagent for total RNA to a 400 μL of exosome suspension, homogenated the mixture, and kept it at rest for 5 min. Then, all subsequent steps were processed on ice. Next, 200 μL of precooled chloroform was added to the solution, and the solution was shaken evenly and allowed to stand for 5 min. After that, the mixture was centrifuged at 4 ℃ for 15 min at 12000 rpm. Next, the upper aqueous phase was carefully transferred to a fresh tube, and an equal volume of precooled isopropanol was added to the supernatant. The mixture was held at −20 ℃ for 30 min to precipitate the total RNA, followed by 10-min centrifugation at 4 ℃ and 12000 rpm. The precipitate was cleaned by adding 1 mL of 75% ethanol and centrifugated at 4 ℃ for 10 min at 12000 rpm. Then, the supernatant was discarded, and the retained precipitation was dried at room temperature for 15 min. The precipitation was dissolved in 40 μL of RNase-free water. The concentrations of the isolated RNA were determined using a NanoDrop One microvolume UV–Vis spectrophotometer.

The total RNA of each clinical sample was divided equally into two and analyzed using the proposed SPCE/ZrGA platform and qRT-PCR (for comparison). The miRNA was reverse transcribed into cDNA using an RT kit (Sangon, China), following a stem-loop-mediated method. The transcribed cDNA was then employed as the template for qPCR using a 2 × SG Fast qPCR Master Mix kit (Sangon, China). The error bars represent the standard deviations of three measurements (n = 3).

All values are presented as the mean ± standard deviations of independent experiments. The results were analyzed by an unpaired, two-tailed Student’s t-test (two groups) or ANOVA (three or more groups) followed by Bonferroni’s correction if needed. All of the statistical tests were performed with GraphPad Prism software version 8.0, and P < 0.01 was considered statistically significant.

# Figures

**Figure S1**

**
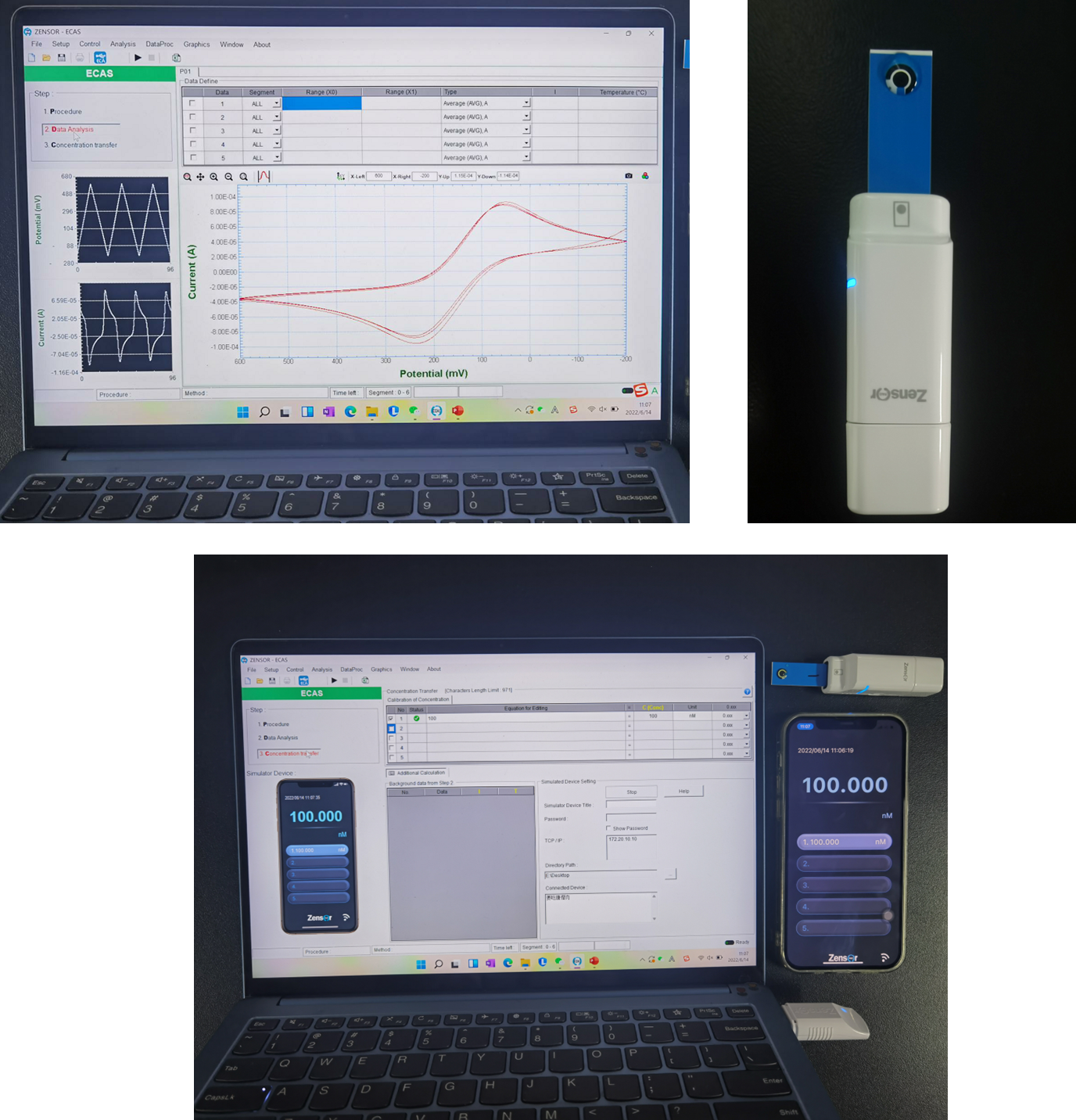
**

**Figure S1.** The detection images of the portable SPCE/ZrGA/mDNA-J bioplatform with a finger-sized U-disk wireless electrochemical analyzer.

**Figure S2**


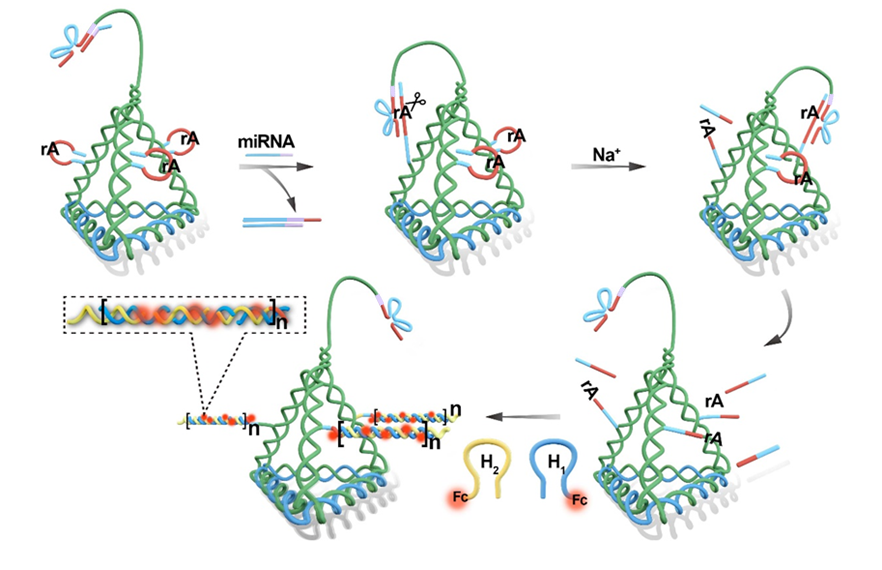


**Figure S2.** Reaction principle of miRNA with mDNA-J**.**

Na^+^-specific DNAzyme was prepared by coassembling the Na^+^ domain, hairpin substrate strands, and enzyme strands, which were inactivated by their locking strands (Lock) and the secondary structure of the hairpin substrate strands. Hairpin substrate strands have a single RNA linkage (rA, ribo-adenine), which serves as a cleavage site. The enzyme strand binds to the substrate via two base-paired regions (red double chain) to form an enzyme precursor (preenzyme). To increase the accessibility of the substrate strands to enzyme strands, a 25-thymine (T) spacer was introduced to the top of the DNA tetrahedron. Na^+^ was used for the catalytic cleavage as the DNAzyme-specific cofactor, which was designed to recognize and cleave the rA site by the formation of a preenzyme structure. The Lock comprises the complementary DNA sequences of the target miRNA-21 and a part of the Na^+^-specific DNAzyme. In the locking strands, a 10-nt DNA domain at the 3'-end was used as a toehold, which could sense the target miRNA and initiate strand displacement between the target miRNA and the enzyme strand. Therefore, in the absence of target miRNA, the substrate-cleaving ability of Na^+^-specific DNAzyme was locked owing to its hybridization with the locking strands. In the presence of target miRNA, the locking strands could sense and hybridize with the target miRNAs and release enzyme strands to open the hairpin structure, then cleave their corresponding substrates, leaving a sticky end to induce the hybridization chain reaction (HCR). Two DNA hairpins (H1 and H2) were labeled with ferrocene (Fc), and the hairpins cannot hybridize with each other without a sticky end. In the presence of sticky-end binding on the edge of the DNA tetrahedron, HCR is induced with a cascade of hybrid events, and the concentration of Fc can be increased by adding H1 and H2.

**Figure S3**


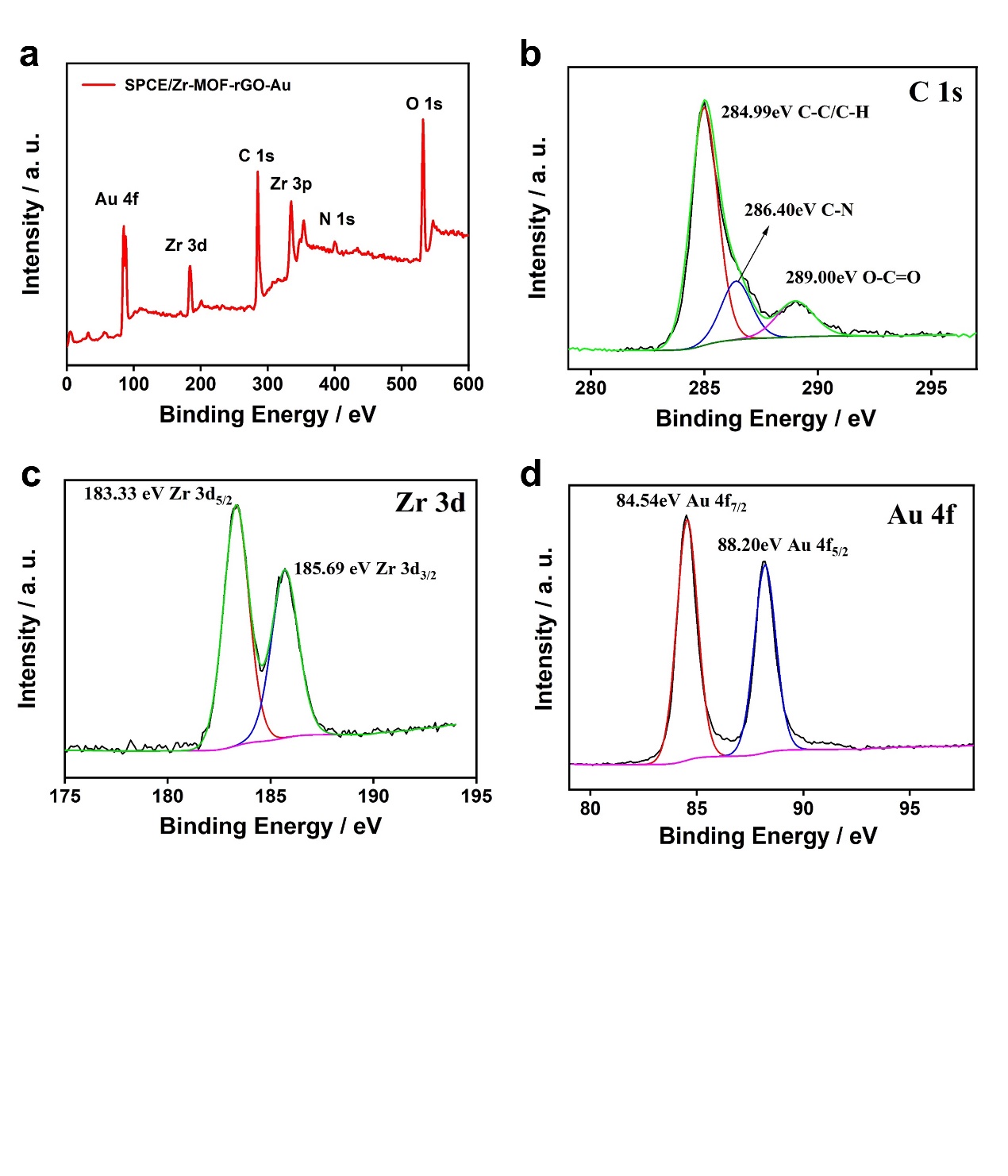


**Figure S3.** (a) Wide-scan XPS of SPCE/ZrGA. High-resolution XPS spectra of C 1s (b), Zr 3d (c), and Au 4f (d) for SPCE/ZrGA.

**Figure S4**


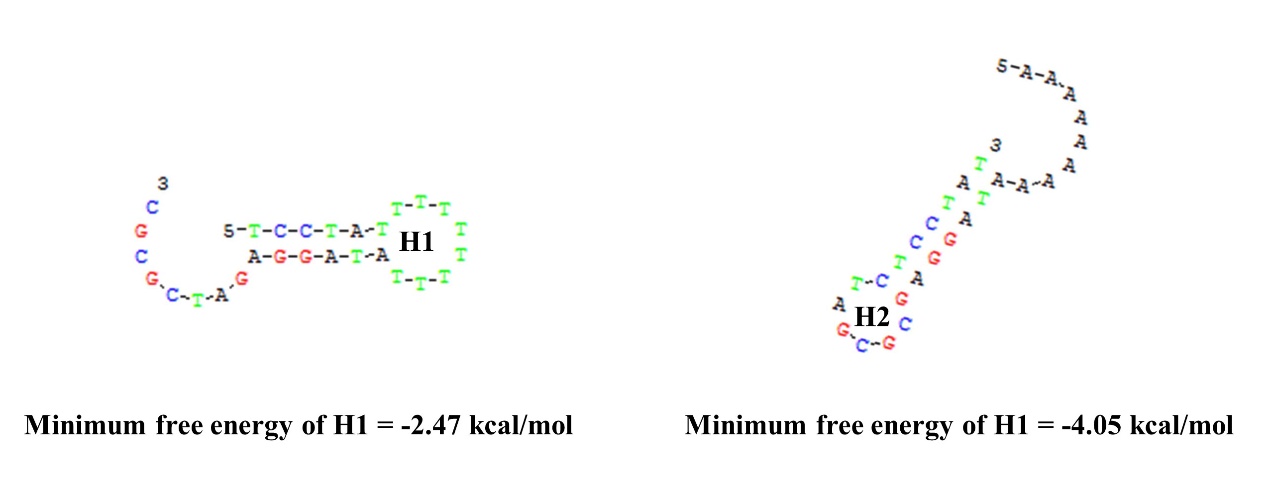


**Figure S4.** Hairpin structures of H1 and H2. The sequences were verified using DNA Man software to ensure the desired hybridization events and avoid the second structure of nucleic acid.

**Figure S5**


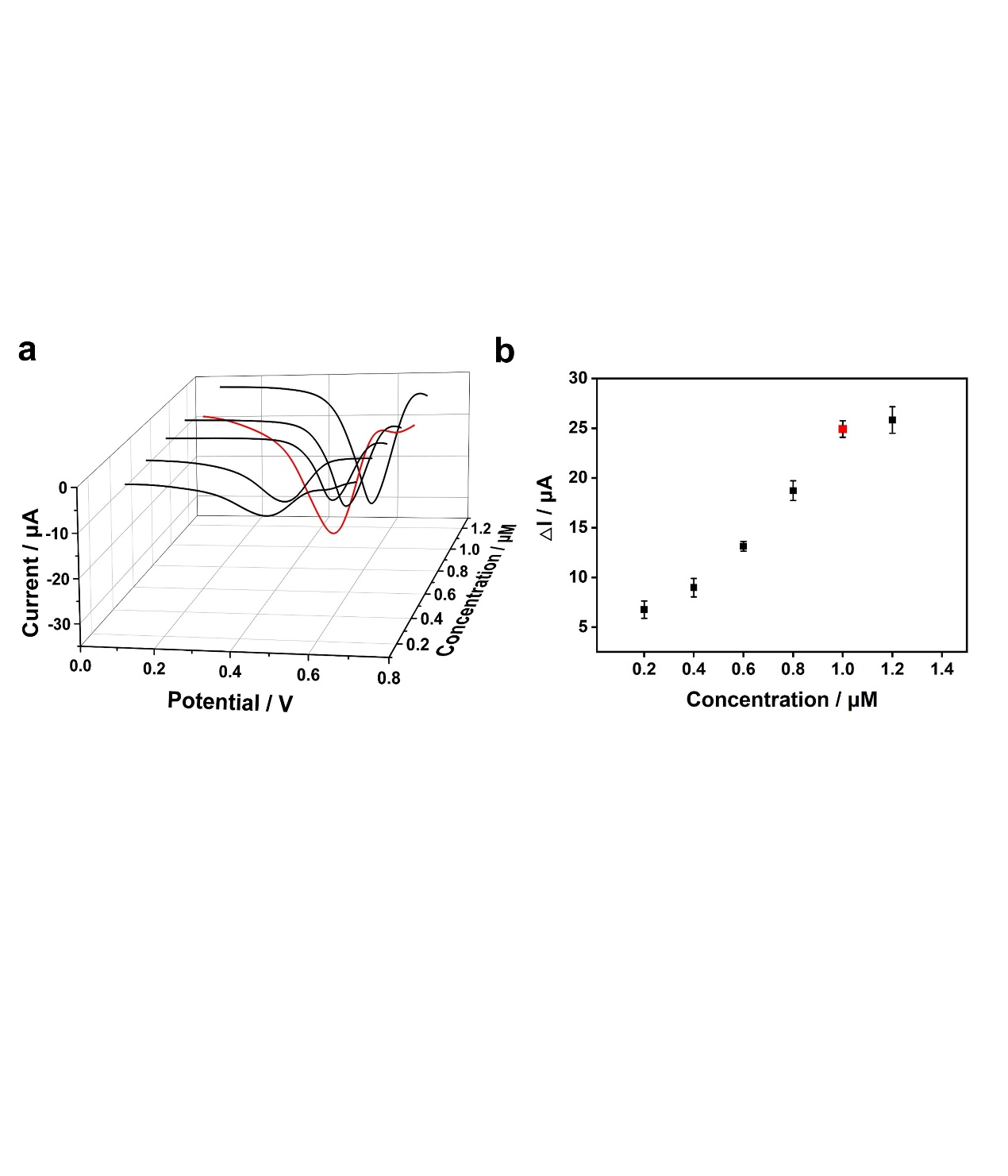


**Figure S5.** Optimization of conditions: (a) SWV response of different mDNA-Js concentrations modified on SPCE/ZrGA and (b) the corresponding scatter plot.

**Figure S6**


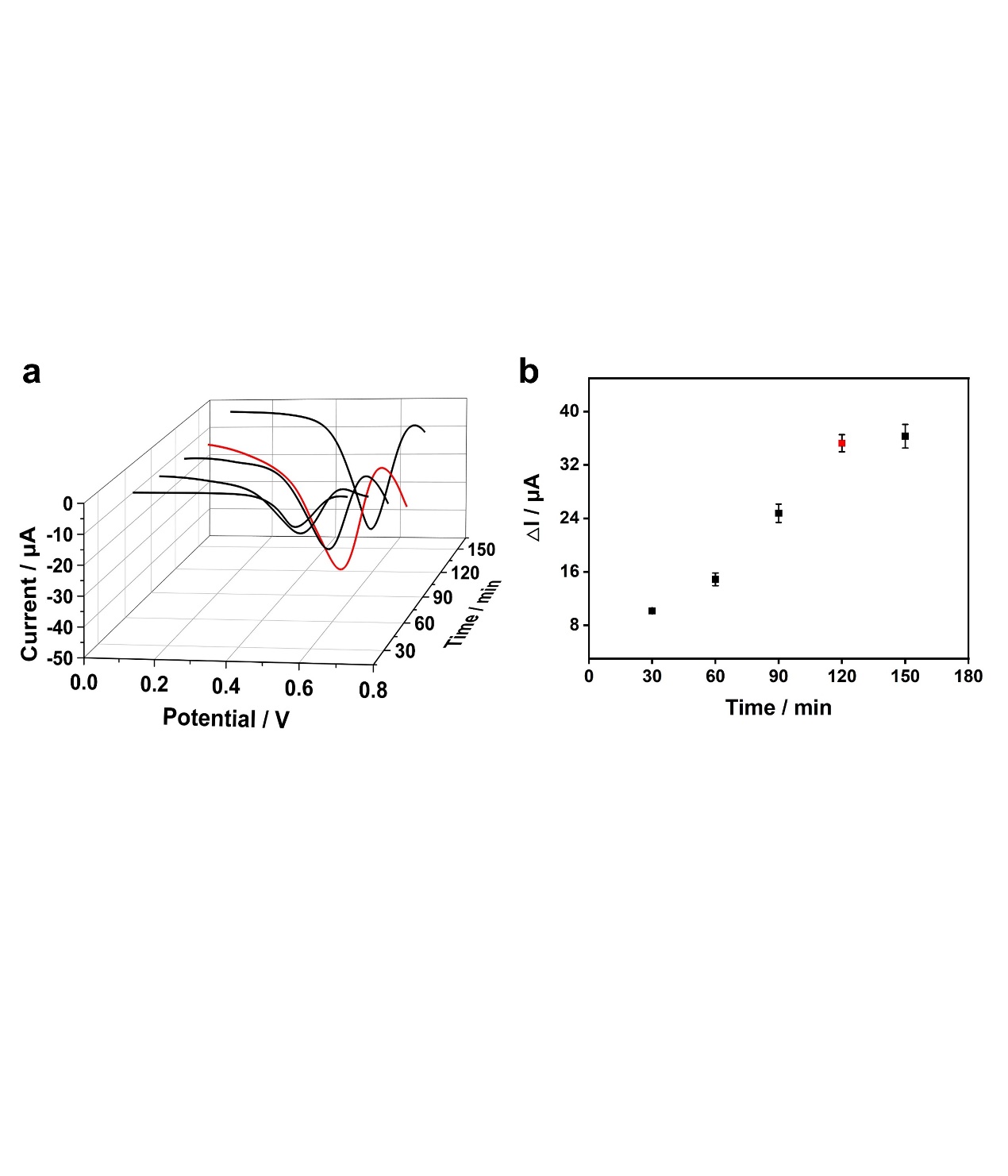


**Figure S6** Optimization of conditions: (a) SWV response of different mDNA-Js incubation times immobilized on the sensing surface of SPCE/ZrGA and (b) the corresponding scatter plot.

**Figure S7**


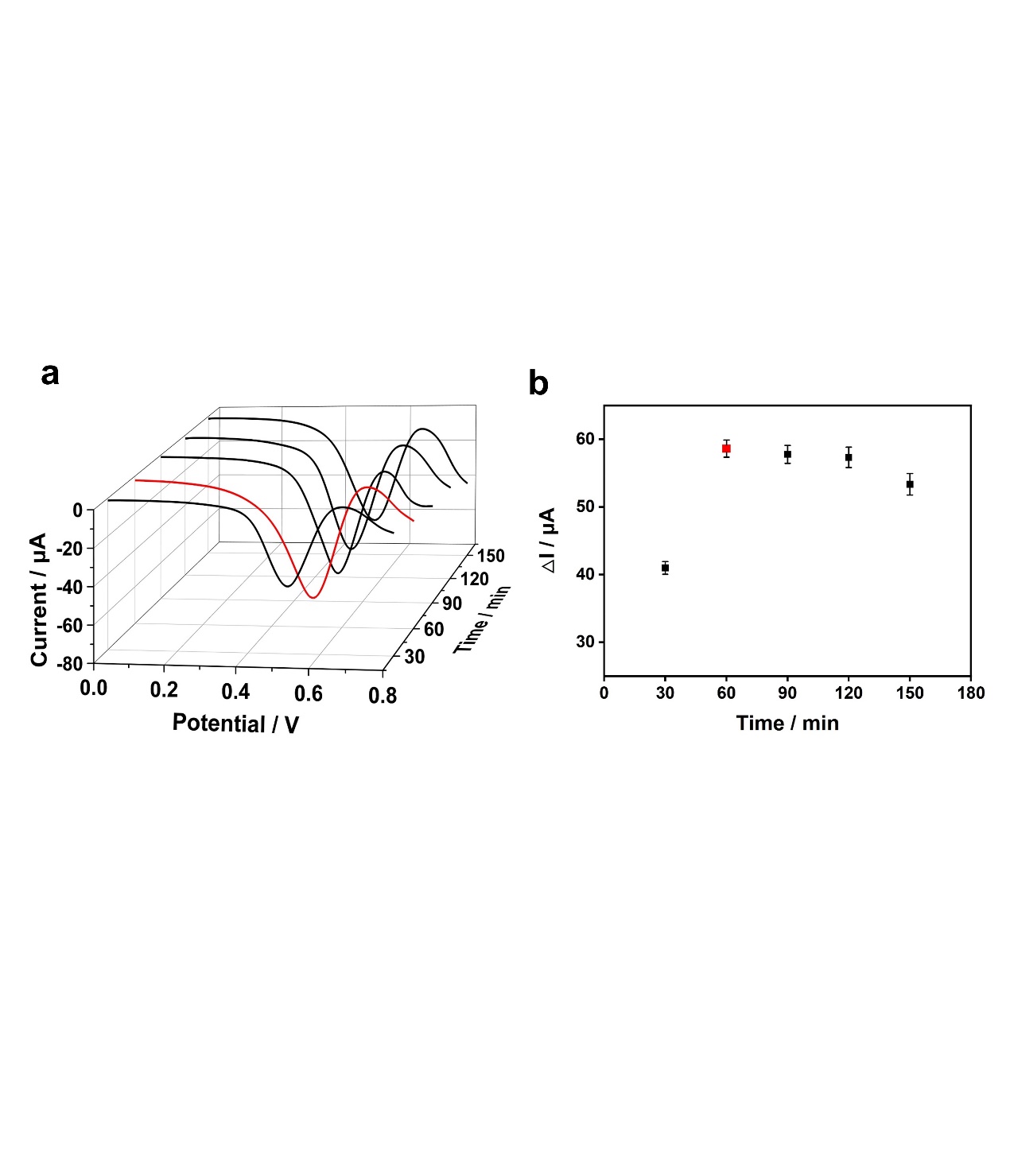


**Figure S7.** Optimization of conditions: (a) SWV response of HCR reaction time (H1 and H2) on the sensing surface of SPCE/ZrGA/mDNA-Js/miRNA and (b) the corresponding scatter plot.

**Figure S8**


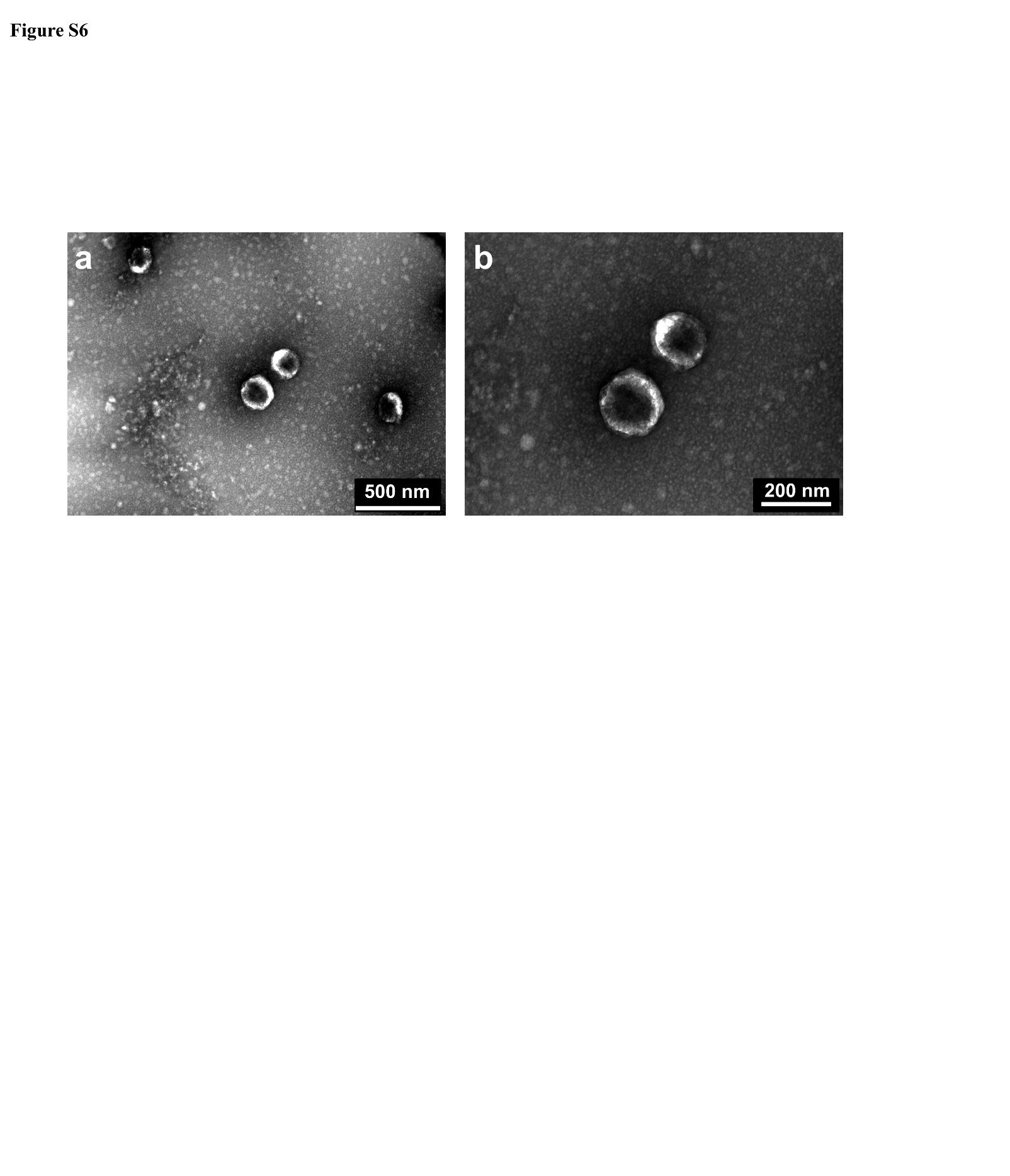


**Figure S8.** TEM image of isolated small exosomes (sEVs) derived from clinical blood samples. The scale bar is 500 nm (a) and 200 nm (b).

**Figure S9**


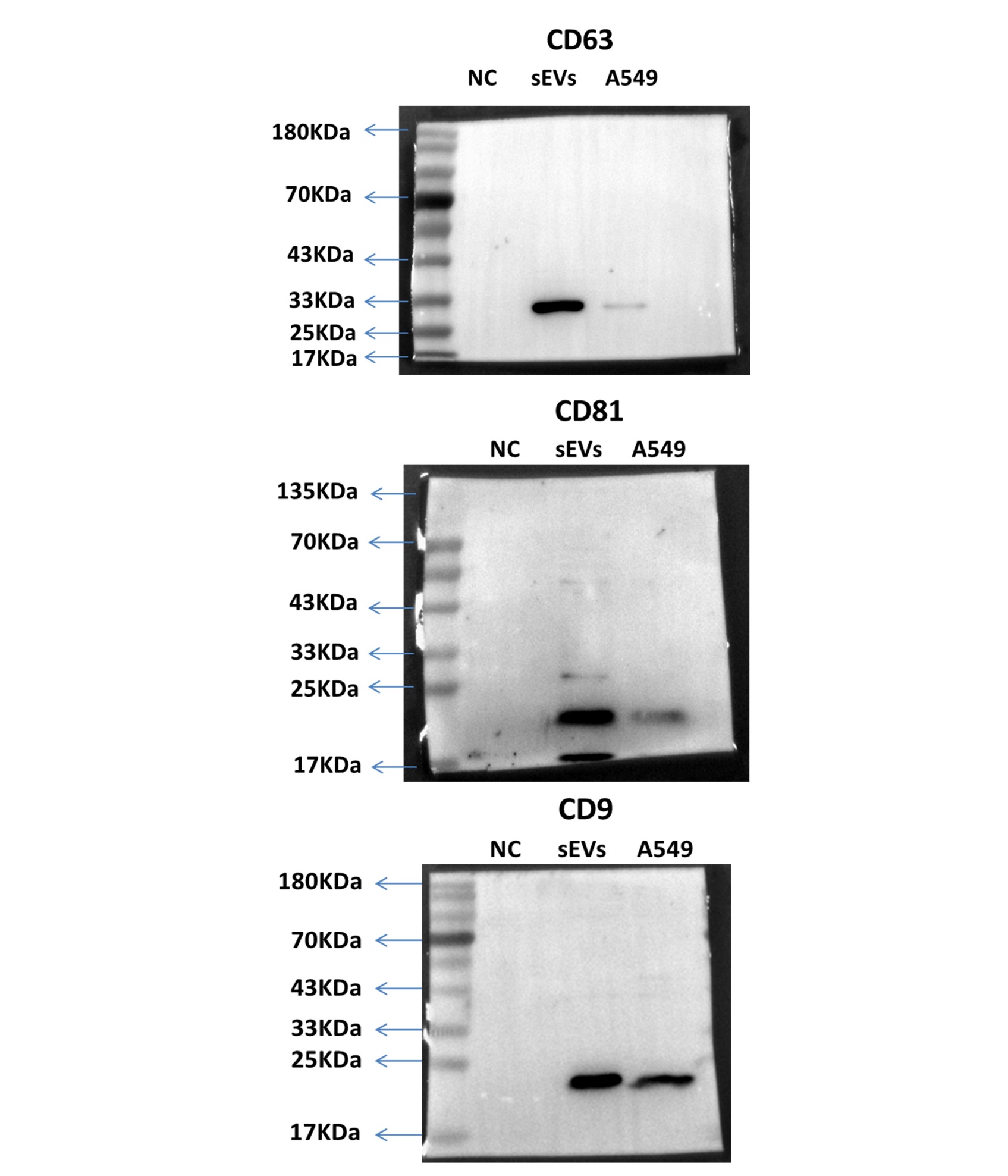


**Figure S9.** The underlying raw images of western blotting analysis (several representative proteins: CD63, CD81, and CD9) of NC, sEVs and A549 cells, NC: PBS.

Western blotting confirmed that the levels of several EV-speciffc markers (CD63, CD81, and CD9) were slightly higher in the extracted sEVs from clinical blood samples (non-small-cell lung cancer (NSCLC) patients) than in A549 cells (from a NSCLC cell line).

# TABLES

**Table S1.** Detailed sequences of the simultaneous detection of miR-21

| **Oligo_name** | **Sequence (5'-3’)** |
| --- | --- |
| S1-a-_SH_ | GTAACAGATAGGCCAATGCTCACGGCGGAGATTACGACGAGACACATGGGAA- HS-SH C6 |
| S_1-r-b_ | CCGAACAATGTTTTCGCGCGATCTCCTAT(rA)  GGAAGTTCCGCCCGCG |
| S_2-a_-_SH_ | AGCATTGGCCATGAAACTCCCAGCGAGCAGCAGGAACGTCCGAATGATATAAA- HS-SH C6 |
| S_2-r-b_ | CTCCGCCGTGTTTTCGCGCGATCTCCTAT(rA)  GGAAGTTCCGCCCGCG |
| S_3-a_-_SH_ | GGGAGTTTCAAGCCGTTCCGACATTGTTCGGAGTAGAACTTAACAGGGAAGCA- HS-SH C6 |
| S_3-r-b_ | GCTGCTCGCTTTTTCGCGCGATCTCCTAT(rA)  GGAAGTTCCGCCCGCG |
| S_4_ | TTATATCATTCGGACGTTCCAGCTTCCCTGTTAAGTTCTACATCCCATGTGTCTCGTCGTAA |
| S_W_ | TCGGAACGGCTTATCTGTTACTTTTTTTTTTTTTTTTTTTTTTTTTTTTTGATGTTGAGGCGGAACCAGGTCAAAGGTGGGTGAGGGGACGCCAAGAGTCCCCGCGGTTAGGAGATCG |
| Lock | TTCCGCCTCAACATCAGTCTGATAAGCTA |
| H_1_-Fc | TCCTATTTTTTTTTATAGGAGATCGCGC-Fc |
| H_2_-Fc | Fc-AAAAAAAAATAGGAGCGCGATCTCCTAT |
| miRNA-21 | UAGCUUAUCAGACUGAUGUUGA |
| miRNA-21-SM | UAACUUAUCAGACUGAUGUUGA |
| miRNA-21-TM | UAACUUACCAGACUGCUGUUGA |
| miRNA-155 | UUAAUGCUAAUCGUGAUAGGGGU |
| miRNA-26a | UUCAAGUAAUCCAGGAUAGGCU |
| miRNA-192 | CUGACCUAUGAAUUGACAGCC |
| miRNA-10b | UACCCUGUAGAACCGAAUUUGUG |

**Table S2.** Detailed sequences of qRT-PCR.

| **Oligo_name** | **Sequence (5'-3’)** |
| --- | --- |
| U6-RT | GTCGTATCCAGTGCAGGGTCCGAGGTATTCGCACTGGATACGACAAAATA |
| U6-F | AGAGAAGATTAGCATGGCCCCTG |
| U6-R | ATCCAGTGCAGGGTCCGAGG |
| miR-21RT | GTCGTATCCAGTGCAGGGTCCGAGGTATTCGCACTGGATACGACTCAACA |
| miR-21 F | AAGAGCGTTAGCTTATCAGACTG |
| miR-21 R | CAGTGCAGGGTCCGAGGT |

**Table S3.** Comparison of the electron transfer rate constant (*k*_s_) obtained here with reported values

| **Modified electrode** | ***k_s_* (s^-1^)** | **Reference** |
| --- | --- | --- |
| Au colloid–cysteamine-modiﬁed gold electrode | 0.490 | ^2^ |
| MWCNT/EmimBF_4_-modified electrodes polyvinyl alcohol film | 1.054 | ^3^ |
| Gold nanoparticles loaded by amino functionalized graphene sheet modified glassy carbon electrode | 1.361 | ^4^ |
| AuNPs/MoS2-rGO modified Glassy Carbon Electrode | 1.201 | ^5^ |
| 3D graphene aerogel- AuNPs modified Glassy Carbon Electrode | 0.56 | ^6^ |
| Zr MOF-rGO-AuNPs/ mDNA-Js modified SPCE | 1.243 | This work |

**Table S4.** Comparison of the analytical performance of the proposed portable biosensor with that of biosensors for miRNA detection

| **Methods** | **Target** | **linear range** | **LOD** | **Refs** |
| --- | --- | --- | --- | --- |
| Electrochemistry | miR-21 | 100 aM-100 pM | 39 aM | ^7^ |
| Electrochemistry | miR-21 | 1 pM−10 nM | 0.26 pM | ^8^ |
| PEC | miR-21 | 1 fM−1 nM | 0.41 fM | ^9^ |
| SERS | miR-155 | 100 fM−5 nM | 1.45 fM | ^10^ |
| ECL | miR-122 | 100 aM−1 pM | 82 aM | ^11^ |
| ECL | miR-21 | 1 fM−1 nM | 0.29 fM | ^12^ |
| ECL | miR-17 | 1 fM-100 pM | 1 fM | ^13^ |
| Fluorescence | miR-21/miR-122 | 50 fM – 500 pM | 20 fM | ^14^ |
| Electrochemistry | miR-21 | 100 aM–0.2 μM | 34.6 aM | This work |

PEC, Photoelectrochemical; SERS, Surface enhanced raman spectroscopy; ECL, Electrochemiluminescence.

**REFERENCES**

1. K. Jayaramulu, M. Horn, A. Schneemann, H. Saini, A. Bakandritsos, V. Ranc, M. Petr, V. Stavila, C. Narayana, B. Scheibe, S. Kment, M. Otyepka, N. Motta, D. Dubal, R. Zboril and R. A. Fischer, *Adv. Mater.*, 2021, **33**, e2004560.

2. Y. Jiang, C. Liu, J. Caro and A. Huang, *Micropor. Mesopor. Mat.*, 2019, **274**, 203-211.

3. M. J. A. Shiddiky and A. A. J. Torriero, *Biosens. Bioelectron.*, 2011, **26**, 1775-1787.

4. F. Pei, P. Wang, E. Ma, H. Yu, C. Gao, H. Yin, Y. Li, Q. Liu and Y. Dong, *Biosens. Bioelectron.*, 2018, **122**, 231-238.

5. J. Dong, L. Wen, H. Yang, J. Zhao, C. He, Z. Hu, L. Peng, C. Hou and D. Huo, *Anal. Chem.*, 2022, **94**, 5846-5855.

6. Y. N. Zhao, Y. Hu, J. Z. Hou, Z. M. Jia, D. Q. Zhong, S. Y. Zhou, D. Q. Huo, M. Yang and C. J. Hou, *J. Electroanal. Chem.* , 2019, **842**, 16-23.

7. P. Miao and Y. Tang, *Small*, 2020, **16**, 2004518.

8. D. Zhu, W. Liu, D. Zhao, Q. Hao, J. Li, J. Huang, J. Shi, J. Chao, S. Su and L. Wang, *ACS Appl. Mater. Inter.*, 2017, **9**, 35597-35603.

9. Y. Zhao, J. Xiang, H. Cheng, X. Liu and F. Li, *Biosens. Bioelectron.*, 2021, **194**, 113581.

10. C.-j. Wu, S.-q. Huang, Y.-y. Wang, Y.-q. Chai, R. Yuan and X. Yang, *Anal. Chem.*, 2021, **93**, 11019-11024.

11. Z.-H. Xu, H. Gao, N. Zhang, W. Zhao, Y.-X. Cheng, J.-J. Xu and H.-Y. Chen, *Anal. Chem.*, 2021, **93**, 1686-1692.

12. X. Y. Li, X. M. Li, D. D. Li, M. Zhao, H. P. Wu, B. Shen, P. Liu and S. J. Ding, *Biosens. Bioelectron.*, 2020, **168**.

13. T. Zhou, R. Huang, M. Q. Huang, J. J. Shen, Y. Y. Shan and D. Xing, *Adv. Sci.*, 2020, **7**, 1903661.

14. H. D. Zhang, X. D. Huang, J. W. Liu and B. H. Liu, *Chem. Sci.* , 2020, **11**, 3812-3819.
